# Fabrication and performance of Zinc-based biodegradable metals: From conventional processes to laser powder bed fusion

**DOI:** 10.1016/j.bioactmat.2024.07.022

**Published:** 2024-07-25

**Authors:** Aobo Liu, Yu Qin, Jiabao Dai, Fei Song, Yun Tian, Yufeng Zheng, Peng Wen

**Affiliations:** a State Key Laboratory of Clean and Efficient Turbomachinery Power Equipment, Department of Mechanical Engineering, Tsinghua University, Beijing, 100084, China; b School of Materials Science and Engineering, Peking University, Beijing, 100871, China; c Department of Orthopedics, Beijing Tsinghua Changgung Hospital, Beijing, 102218, China; d Department of Orthopedics, Peking University Third Hospital, Beijing, 100191, China

**Keywords:** Biodegradable metal, Zinc, Additive manufacturing, Laser powder bed fusion, Porous scaffolds

## Abstract

Zinc (Zn)-based biodegradable metals (BMs) fabricated through conventional manufacturing methods exhibit adequate mechanical strength, moderate degradation behavior, acceptable biocompatibility, and bioactive functions. Consequently, they are recognized as a new generation of bioactive metals and show promise in several applications. However, conventional manufacturing processes face formidable limitations for the fabrication of customized implants, such as porous scaffolds for tissue engineering, which are future direction towards precise medicine. As a metal additive manufacturing technology, laser powder bed fusion (L-PBF) has the advantages of design freedom and formation precision by using fine powder particles to reliably fabricate metallic implants with customized structures according to patient-specific needs. The combination of Zn-based BMs and L-PBF has become a prominent research focus in the fields of biomaterials as well as biofabrication. Substantial progresses have been made in this interdisciplinary field recently. This work reviewed the current research status of Zn-based BMs manufactured by L-PBF, covering critical issues including powder particles, structure design, processing optimization, chemical compositions, surface modification, microstructure, mechanical properties, degradation behaviors, biocompatibility, and bioactive functions, and meanwhile clarified the influence mechanism of powder particle composition, structure design, and surface modification on the biodegradable performance of L-PBF Zn-based BM implants. Eventually, it was closed with the future perspectives of L-PBF of Zn-based BMs, putting forward based on state-of-the-art development and practical clinical needs.

## Highlights

• Recent advances in L-PBF Zn-based BMs, including Zn, Zn alloys, and Zn-based composites, were comprehensively reviewed.
• Required properties of L-PBF Zn-based BMs for medical implants were summarized.
• The impact of powder composition, structure design, and surface modification on biodegradable performance was clarified.
• Future perspectives for L-PBF Zn-based BMs were presented.

## Graphical abstract

**Figure:**
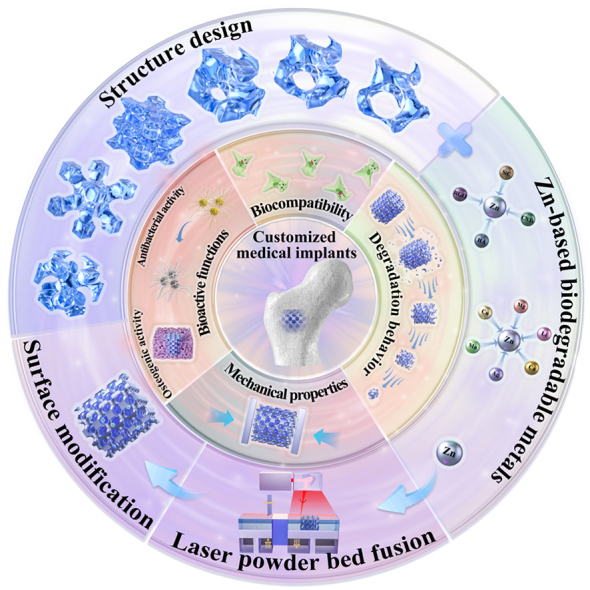

**Table:** List of abbreviations

3D	Three-dimensional
ALD	Atomic layer deposition
ALP	Alkaline phosphatase
AM	Additive manufacturing
BD	Building direction
BJ	Binder jetting
BM	Biodegradable metal
CAD	Computer-aided design
CNF	Carbon nanofiber
CNT	Carbon nanotube
CS	Compressive strength
CYS	Compressive yield strength
DED	Direct energy deposition
E	Elastic modulus
*E. coli*	Escherichia coli
GA	Gas atomization
GBR	Guided bone regeneration
GNS	Graphene nanosheets
HA	Hydroxyapatite
L-PBF	Laser powder bed fusion
mBMSC	Mouse bone marrow mesenchymal stem cell
PBF	Powder bed fusion
PDA	Polydopamine
RD	Relative density
RE	Rare earth
RGO	Reduced graphene oxide
r-SBF	Revised simulated body fluid
*S. aureus*	Staphylococcus aureus
SD rat	Sprague-Dawley rat
SMC	Smooth muscle cell
SPE	Single point exposure
TI	Texture intensity
TPMS	Triply periodic minimal surface
TYS	Tensile yield strength
UTS	Ultimate tensile strength
Van	Vancomycin
WA	Water atomization
β-TCP	Beta-tricalcium phosphate

## 1 Introduction

AM creates 3D objects by depositing materials layer by layer, providing a reliable way to obtain complicated 3D structures [1]. Compared with traditional manufacturing methods, AM has the advantages of free structural design, less material waste, and short lead time, and it has been successfully applied in fields such as aerospace, medicine, and personalized consumer goods [[2], [3], [4]]. According to the ISO 52900 standard, AM technologies can be divided into seven families: vat photopolymerization, PBF, BJ, material jetting, sheet lamination, material extrusion, and DED [1]. BJ, DED, and PBF are the main methods for manufacturing metallic parts [5,6]. However, not all these three technologies are fit for the fabrication of clinical implants. For BJ, it isn't easy to guarantee the densification and mechanical properties of the product [7]. DED is suitable for manufacturing large components with high efficiency but not for implants with strict requirements for delicate structures and dimensional accuracy [8]. Compared with DED, PBF has higher formation accuracy due to its smaller beam spot, finer powder particles, and thinner layer thickness, making it more suitable for fabricating implants. In the PBF process, the powder is spread as a thin layer on the platform in advance. High energy density heat sources, including laser and electron beams, selectively melt powder layers according to computer-aid design. The process is repeated layer by layer until the designed components are successfully manufactured [[8], [9], [10], [11]].

PBF has successfully achieved the reliable fabrication of non-degradable metals, such as Ti alloys, Ta alloys, stainless steels, etc. It has been developed into the most widely used AM method for producing metallic implants [11]. However, Zn-based BMs have a high vaporization tendency. The melting of such powders by laser or electron beams will produce massive evaporation fume. The beam propagation can be seriously scattered by the tiny particles of the fume, resulting in unstable melting. Much worse, the fume can pollute the chamber and damage the machine. Accordingly, it is a critical issue to eliminate the negative effect of evaporation fume in the PBF processing chamber. Electron beam PBF necessitates operation within a vacuum environment, making it difficult to process metals of high vaporization tendency. L-PBF is used most for AM of Zn-based BMs [9]. In 2016, the first peer-review work on L-PBF of pure Zn was published, verifying the feasibility of producing Zn-based BMs by AM [12].

The L-PBF of Zn-based BMs is expected to solve the dual challenges of customized structure and biodegradable performance of implants, and has attracted increasing attention as the future of medical device manufacturing. In the latest five years, the L-PBF of Zn-based BMs has developed rapidly for biodegradable applications [13]. Researchers have continuously optimized the composition of powder particles, structure design of implants and L-PBF processes for reliable fabrication and satisfactory properties. Zn alloys, such as Zn–Mg, Zn–Li, and Zn–Ce, as well as Zn-based composites, using reinforcements like nano-SiC, RGO, and CNTs, have been successfully manufactured by L-PBF processes [[14], [15], [16], [17], [18], [19]]. Porous scaffolds with customized structures were fabricated with high formation quality [20,21]. *In vitro* and *in vivo* tests of Zn-based BM implants have demonstrated encouraging results [22,23]. Benefiting from L-PBF, more possibilities are expected for future clinical applications with Zn-based BM implants of customized compositions and structures.

This paper reviewed the research status of L-PBF Zn-based BMs, including pure Zn, Zn alloys, and Zn-based composites. Powder particles, structure design, processing optimization, chemical compositions, surface modification, microstructure, mechanical properties, degradation behaviors, biocompatibility, and bioactive functions were discussed and analyzed. The influence of powder particle composition, structure design, and surface modification on the biodegradable performance of implants was clarified. The current development and future prospective on Zn-based BM implants fabricated by L-PBF were summarized in the end.

## 2 Zn-based BMs fabricated by conventional processes

BMs can be gradually corroded in body fluids with appropriate host responses elicited by released corrosion products, which can pass through or be metabolized or assimilated by cells and/or tissues. Remarkably, BMs fully dissolve and leave no implant residue after they fulfill their role in aiding tissue healing [24,25]. The advancement of BMs holds significant value in the medical field as they reduce the risk of infection associated with long-term implants and eliminate the necessity for subsequent surgeries. Notably, in load-bearing medical applications such as bone implants, BMs offer a solution to the problem of insufficient mechanical strength presented by the current biodegradable materials including polymers and ceramics [9]. In addition to appropriate mechanical properties, ideal medical implants should also demonstrate excellent biocompatibility, controlled degradation, and specific bioactive functions to ensure effective treatment. As a result, research on the performance of BMs primarily focuses on the above four fundamental aspects, which are majorly decided by composition and manufacture [25]. Various Zn-based BM raw bulks have been successfully produced by forming processes, such as casting, powder metallurgy and transient directional solidification. Various fabrication techniques, including extrusion, drawing, rolling, forging and so on, have been further employed to acquire the final shape as well as to modulate microstructure [26]. This section mainly focuses on the performance and promising medical applications of Zn-based BMs fabricated by conventional processes.

### 2.1 Biocompatibility

The concept of “biocompatibility” refers to an implant material's capacity to perform effectively in the body without inducing any adverse local or systemic reactions. Assessing the biocompatibility of medical devices is a critical step in obtaining regulatory approval [27,28]. Consequently, a significant portion of research concentrates on the evaluation of the biocompatibility of BMs. In contrast to non-degradable metals, BMs are made up of elements that can be metabolized by the human body, and demonstrate appropriate degradation rates and modes. In terms of the mass ratio within the human body, life-related metal elements are ranked as follows: Ca, K, Na, Mg, Fe, and Zn. Since Ca, K, and Na are too active to be used as structural materials, the focus of BMs has been placed on Mg, Fe, and Zn-based BMs [25].

Cytocompatibility and hemocompatibility are widely used to assess the biocompatibility of BMs. Cells are the basic unit of function in living organisms, and cytotoxicity is a preferred item in biological tests to provide prima facie evidence for the identification and assessment of other biological sources of risk [29]. The cytocompatibility of Zn is found to be dose-dependent. For human SMCs, a low concentration of Zn^2+^ (<80 μM) enhanced cell adhesion, spreading, proliferation and migration, and boosted the expression of actin and vinculin. Conversely, at a high Zn^2+^ concentration around 80–120 μM, cells exhibited opposite cellular responses and behaviors [30]. Yang et al. reported that the cell viability in 100 % Zn extracts was lower than 20 % for mouse osteoblastic cells (MC3T3-E1), indicating severe cytotoxicity [31]. This suggests that the cytocompatibility of pure Zn requires improvement for clinical applications. In response, various of alloys and composites have been invented to improve the cytocompatibility of Zn-based BMs.

**Fig. 1:**
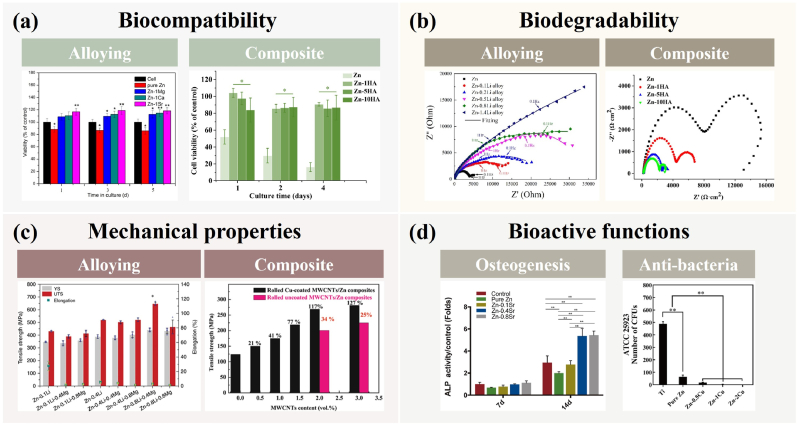
Required properties of Zn-based BMs for medical implants: (a) biocompatibility: MG63 cell viability cultured in cell culture medium, 100 % pure Zn and Zn alloy extracts for 1, 3, and 5 days [32]; Cell viability of MC3T3-E1 cells after cultured in 100 % Zn and Zn-HA sample extracts for 1, 2 and 4 days (adapted from Ref. [36]), (b) biodegradability: Nyquist plots of Zn and Zn–Li alloys [45]; Nyquist plots of Zn and Zn-HA composites [36], (c) mechanical properties: tensile properties of Zn, Zn–Li, and Zn–Li–Mg [31], tensile strength of CNTs/Zn composites [55], (d) bioactive functions: osteogenesis: ALP activity of Zn and Zn–Sr alloys (extracts diluted 1-fold) [61], anti-bacteria: Colony counts of ATCC25932 bacteria on Ti, Zn, and Zn–Cu alloy surfaces after 1 day of culture [65].

Cytocompatibility of Zn alloys with Mg, Ca, Sr, Li, Mn, Fe, Cu, Ag and RE elements was experimentally investigated [[31], [32], [33], [34], [35]]. With the addition of 1 wt% nutrient alloying elements, Li et al. discovered that binary alloys Zn–1Mg, Zn–1Ca and Zn–1Sr all showed improved cell viability using human osteosarcoma MG63 cells and human umbilical vein endothelial cells (ECV304), as shown in Fig. 1a [32]. Sun et al. found that Zn-0.5Li alloy exhibited less cytotoxicity to MC3T3-E1 cells compared to pure Zn, attributed to the reduced release of Zn^2+^ due to the preferential release of Li^+^ [33]. Jia et al. reported that Zn–Mn alloys showed enhanced cell viability compared with pure Zn, as trace amounts of Mn^2+^ positively influenced cytocompatibility [34]. The cytocompatibility of Zn-based composites with HA was also investigated. With the addition of HA, cell viability of MC3T3-E1 in Zn-1HA extract exceeded 100 % on the first day, significantly higher than the 51.99 % observed in pure Zn extraction (Fig. 1a) [36]. Furthermore, in addition to cytocompatibility, the hemocompatibility of Zn-based BMs was evaluated. Hemocompatibility is the ability of blood to respond appropriately to exogenous substances or materials. Satisfied hemocompatibility is a prerequisite for biomaterials to be used as medical implants [37]. Pure Zn displayed a hemolysis rate of less than 5 %, designating it as blood-compatible according to the ISO 10993-4 standard. Zn alloys and composites, such as Zn-RE, Zn-0.8Li, and Zn-HA, also demonstrated hemolysis rates of less than 5 %, indicating good blood compatibility [35,36,38].

### 2.2 Biodegradability

BM implants should maintain their support function until the damaged tissues/organs recover the mechanical integrity, and then completely degrade after fulfilling their role in assisting with tissue healing [25,39]. Hence, meticulous attention must be directed towards understanding the degradation behavior of BMs. The primary degradation process of Zn-based BMs is shown in Eqs. (1), (2), (3), (4), (5), (6). Compared with Mg, which primarily undergoes hydrogen precipitation reactions in body fluids, Zn mainly undergoes oxygen reduction reactions. Therefore, no significant gas generation is observed during the *in vitro* and *in vivo* degradation of Zn. The degradation products of Zn-based BMs predominantly include ZnO, ZnCO_3_, Zn_3_(PO_4_)_2_, and trace amounts of calcium and phosphorus salts [40,41]. Given that ZnO exhibits a high solubility product constant and thermodynamic stability within body fluids with pH values ranging from 8 to 13, it effectively protects the matrix, leading to a low degradation rate to a certain degree [42]. The *in vivo* degradation rate of pure Zn is approximately 0.2 mm/year. The complete degradation of a pure Zn screw with a diameter of 5 mm may take more than a decade, which is considerably longer than the 3–6 months typically required for bone healing [36]. Moreover, severe localized corrosion is observed in pure Zn, elevating Zn^2+^ concentrations in the bone environment and potentially impeding bone regeneration. In pursuit of optimized degradation behaviors, various Zn alloys and Zn-based composites have been invented [22,43].

(1) Zn → Zn^2+^ + 2e^−^

(2) O_2_ + 2H_2_O + 4e^−^ → 4OH^−^

(3) Zn^2+^ + 2OH^−^ → Zn(OH)_2_

(4) Zn^2+^ + 2OH^−^ → ZnO + H_2_O

(5) Zn(OH)_2_ + 2Cl^-^ →Zn^2+^ + 2OH^−^ + 2Cl^-^

(6) 3Zn^2+^ + 2HPO_4_^2−^ + 2OH^−^ + 2H_2_O → Zn_3_(PO_4_)_2_·4H_2_O

The degradation behavior of pure Zn can be controlled by alloying. The influence of alloying on degradation is closely related to the type and content of the alloying elements. The microstructural characteristics, including grain size distribution and phase composition, which significantly influence the degradation process, change upon the introduction of alloying elements. Mostaed et al. observed that the corrosion current density of Zn–Mg alloys increased with the content of Mg rising from 0 to 1 wt%. When 3 wt% of Mg was added, the alloy demonstrated enhanced corrosion resistance compared to pure Zn, due to the formation of a uniformly distributed Mg_2_Zn_11_ phase and the stabilizing effect of Mg on the protective layer of Zn [44]. Li et al. developed a series of Zn–Li alloys and found that the corrosion density decreased as the Li content increased from 0.2 to 1.4 wt% (Fig. 1b). The enhanced corrosion resistance of Zn–Li alloys resulted from the formation of a stable Li_2_CO_3_ corrosion product. However, the corrosion rate of Zn-0.1Li was higher than that of pure Zn, attributed to the galvanic corrosion between the LiZn_4_ and Zn matrix [45]. Zou et al. discovered that Zn-xCa alloys (x = 0.5, 1, 2, 3 wt%) all had higher corrosion rates compared to pure Zn, and Zn–2Ca exhibited the highest corrosion rate due to the presence of a large number of two-phase boundaries [46]. Yang et al. assessed the *in vitro* and *in vivo* degradation behaviors of Zn-0.4Gd. The Zn-0.4Gd alloy exhibited a more uniform degradation mode, generating beneficial microenvironments for bone regeneration due to modified grain size distribution and texture when compared to pure Zn [43].

Similar to Zn alloys, the degradation behaviors of Zn-based composites vary depending on the type and content of the doped materials. Yang et al. developed Zn-HA composites and discovered that Zn-5HA and Zn-10HA exhibited accelerated degradation rates, while Zn-1HA presented a slightly reduced degradation rate compared to pure Zn, as shown in Fig. 1b. HA, characterized by low solubility and poor conductivity, obstructed the charge transfer among Zn particles, thereby enhancing corrosion resistance. However, the existence of pores and voids at the interface between Zn and HA resulted in elevated degradation rates. Furthermore, HA located in the particle boundary offered more sites susceptible to corrosion attack. These factors collectively influenced the degradation behavior of Zn-HA composites [36]. Sun et al. found that the addition of β-TCP into pure Zn decreased the corrosion rate and effectively suppressed localized corrosion. This was attributed to the enhanced continuity of the network structure by β-TCP, which impeded corrosion. Moreover, β-TCP could stabilize the grain boundary area, improving the protective effect of corrosion products and thereby inhibiting localized corrosion [47]. Dai et al. developed Zn-GNS composites and found that the corrosion rate increased with a higher GNS content. Mainly uniform corrosion and a little localized corrosion, resulting from the potential difference between GNS and Zn matrix, and the presence of pores and cracks at the interface, were observed during degradation [48].

### 2.3 Mechanical properties

The adequate strength of medical implants is essential to guarantee their functional stability and reduce the risk of fractures after implantation. Furthermore, in the case of orthopedic implants, it is imperative that the elastic modulus of the materials utilized closely approximates that of human bone to mitigate the stress shielding effect [9]. BM implants should have a yield strength greater than 200 MPa [49]. However, the yield strength of cast pure Zn is only 28 MPa. By utilizing hot-extruded manufacturing methods, the yield strength of pure Zn can be improved to 124 MPa due to the grain refinement, which is highest among all pure Zn fabricated by different manufacturing methods, but still insufficient for load-bearing applications. Moreover, Young's modulus of pure Zn is about 97 GPa, greatly higher than that of natural bone (cortical bone: 5–23 GPa, trabecular bone: 0.01–1.6 GPa) [26,50,51]. Therefore, to improve mechanical properties of Zn-based BMs, several attempts have been undertaken. For enhancing strength, alloying and doping reinforcement particles are proven to be effective strategies, as shown in Fig. 1c.

Alloying effectively enhances the strength by solution strengthening, precipitation strengthening, and grain refinement strengthening. Yang et al. evaluated the strengthening effects of eight elements (Mg, Ca, Sr, Mn, Cu, Li, Ag, and Fe) on Zn-based BMs. Their findings indicated that Li, Mg, Cu, Ag, and Mn exhibited significant strengthening effects on pure Zn, with Li and Mg showing the most pronounced strengthening effect. The UTS of hot-extruded Zn-0.4Li and Zn-0.4Mg alloys was 520.36 MPa and 354.89 MPa, respectively [31]. The high strength of the Zn–Li alloy primarily originated from the formation of a β-LiZn_4_/Zn lamellar structure, while the increased strength of the Zn–Mg alloy was due to grain refinement and second-phase strengthening. Upon additional Mg added into the Zn–Li alloy, the UTS of the hot-extruded Zn-0.8Li-0.4 Mg alloy reached 646.69 MPa, equivalent to that of pure Ti and 316 stainless steel (Fig. 1c) [31,52,53]. In recent years, RE elements have also been utilized in the Zn alloys, playing an essential role in the improvement of their mechanical behaviors. Du et al. developed 16 binary Zn-0.2 at% RE alloys and reported their mechanical properties. All Zn-RE alloys exhibited notably higher tensile strength compared to pure Zn. Among these alloys, the hot-extruded Zn–Y alloy had the highest UTS, reaching 219 MPa. Owing to the limited solubility of RE elements in Zn, the alloying elements primarily existed in the form of the second phase. The enhancement in strength primarily resulted from the reinforcement offered by the second phase [35].

In addition to alloying elements, reinforcement materials, such as β-TCP and CNTs, have also been added to pure Zn to improve its mechanical properties. Pan et al. investigated mechanical behaviors of hot-extruded Zn–Mg/β-TCP composites. The composite showed the highest UTS of 330.5 MPa when adding 1 vol% β-TCP. The strengthening effect could be explained by dislocation plugging from the addition of particles. When more particles were added, the strength of Zn-based BMs decreased due to the reunion of particles [54]. Ao et al. reported an enhancement in the strength of Zn-based BMs by adding CNTs, as shown in Fig. 1c. The UTS of hot-rolled Zn/CNTs reached 281 MPa, 127 % higher than that of pure Zn. The primary strengthening mechanisms were grain refinement and the load transfer effect [55].

The development of Zn alloys and Zn-based composites has successfully solved the problem of low mechanical strength of pure Zn and greatly broadened the scope of its application. The Zn-based BMs with optimized composition have a comparable or even higher strength compared with Mg-based BMs. Moreover, Zn-based BMs maintain high strength, with only a slight decrease after eight weeks of *in vitro* immersion [32]. This characteristic ensures stable mechanical support throughout the tissue repair process, making Zn-based BMs more compatible with the mechanical requirements of implant materials. However, it is worth noting that although compositional optimization can effectively enhance the strength of Zn-based BMs, it does not concurrently reduce the elastic modulus. Therefore, how to reduce the elastic modulus of Zn-based BMs is still an urgent problem to be solved.

### 2.4 Bioactive functions

Zn ranks as the second most abundant micronutrient in living organisms, instrumental in protein synthesis, energy metabolism, and multiple enzymatic reactions. It exhibits a wide range of bioactive functions, with a particular focus on its osteogenic and antibacterial ability in the research of Zn-based BM implants, as shown in Fig. 1d [56]. Zn plays an important role in the process of bone formation and mineralization. In the bone environment, Zn in osteoblasts promotes protein synthesis through activation of tRNA synthetases and stimulation of gene expression, as well as increasing the amount of intracellular DNA, thus facilitating bone formation and mineralization. At the same time, Zn promotes apoptosis of osteoclasts by modulating the Ca ion signaling pathway. Zn ultimately increases bone mass by promoting osteogenesis and inhibiting bone resorption [57,58]. Huo et al. reported that the loading of Zn on Ti implants increased their osteogenesis inducing activity [59]. However, this promotion of osteogenesis was found to be dose-dependent. Xiong et al. reported that ALP activity of the mBMSCs and the up-regulation of the gene expression related to osteogenesis were promoted when the concentration of Zn^2+^ was around 10.91–27.15 μM. However, at a high Zn^2+^ concentration (128.58 μM), the ALP activity was lower than that in the control group [60]. Studies have demonstrated that optimizing the composition can endow Zn-based BMs with superior osteogenic abilities. Alloying with Sr resulted in enhanced ALP activity in MC3T3-E1 cells and increased expressions of osteogenesis-related genes, potentially due to the synergistic effects of Zn^2+^ and Sr^2+^ (Fig. 1d) [61]. Yao et al. studied the osteogenic activities of Zn–1Mg-β-TCP composites. The *in vivo* results showed that the osteogenic ability of Zn–1Mg-β-TCP was higher than pure Zn, and increased with the addition of β-TCP, which exhibited good osteoconductivity [62].

In clinical treatments, implant-related infections represent a significant concern. These infections typically originate from free bacteria adhering to implant surfaces, resulting in the formation of a bacterial biofilm that is difficult to remove [63]. Zn-based BMs emerge as a formidable countermeasure to the infection, attributable to the broad-spectrum antibacterial ability of Zn^2+^ [41]. Zn^2+^ interacts with bacterial surfaces, altering the charge balance of bacterial cell membranes and leading to deformation and rupture of the bacteria [64]. The antibacterial properties of Zn-based BMs can be further enhanced by the addition of alloying elements with strong antibacterial activity, such as Cu and Ag. Qu et al. reported that the antibacterial activity of Zn–Cu alloy was significantly higher than that of both pure Zn and pure Ti. Zn–Cu alloys showed strong antibacterial efficacy against coagulase-positive and coagulase-negative staphylococci, and antibiotic-resistant strains (MRSA and MRSE) by preventing bacterial adhesion and biofilm formation. At the genetic level, the expression of genes related to adhesion, biofilm formation, autolysis, virulence and drug resistance in MRSA was affected by Zn–2Cu alloy (Fig. 1d) [65]. Zn–Ag alloys were also investigated by Qu et al. for their antibacterial properties. Compared to pure Zn, Zn–1Ag and Zn–2Ag alloys exhibited superior antibacterial effects, potentially due to the inhibition of biofilm formation, antibiotic resistance pathways, and autolysis-related pathways [66]. Jia et al. reported that bacteriostatic rings of Zn-0.8Li-xAg alloys were larger than those of Zn-0.8Li, indicating improved antibacterial activity after alloying with Ag. The MRSA-killing mechanisms of Zn-0.8Li-0.5Ag alloy were cellular metabolism disturbance and induction of reactive oxygen species production. The inhibited biofilm formation and virulence of MRSA, and alleviated drug resistance were also verified [67].

### 2.5 Promising medical applications

**Fig. 2:**
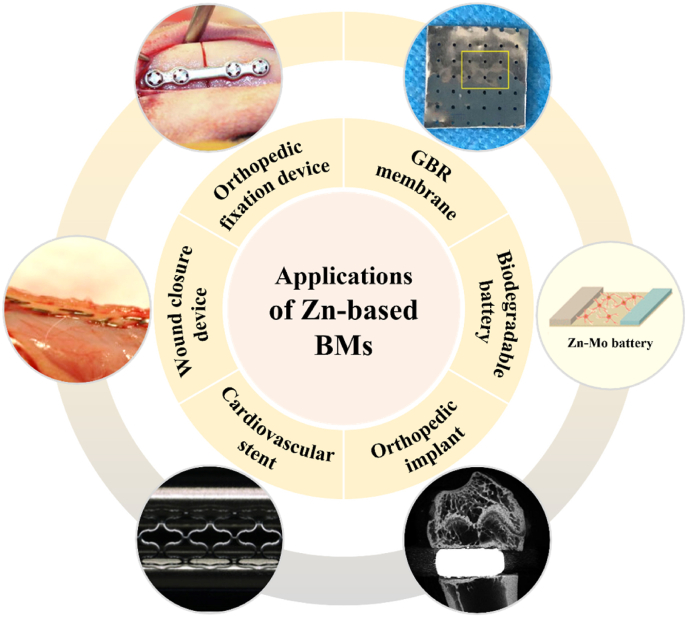
Promising medical applications of Zn-based BMs: wound closure devices [69], orthopedic fixation devices [70], GBR membranes [73], biodegradable batteries [71], cardiovascular stent [76], orthopedic implants [36].

Zn-based BMs are promising materials for various potential clinical applications, such as wound closure devices, orthopedic fixation devices, biodegradable batteries, GBR membranes, cardiovascular stents and bone implants due to their performance in four aspects mentioned above. Polymer staples have been successfully used for wound closure. However, the mechanical strength of the polymer is not sufficient, leading to the rupture of the wound along the surgical incision. Moreover, the polymer is not hard enough to be used in excessively thick tissues. It is also tricky for limb incisions because of the inadequate amount of subcuticular space, which is essential for the slight eversion of the skin after staple attachment. Zn-based BMs provide a way to deal with these issues, because they have higher strength and hardness compared with polymers. The staples can be designed thinner and leave a smaller footprint [68]. Amano et al. investigated the feasibility of Zn alloy staples, as shown in Fig. 2. Zn–Cu–Mn–Ti, Zn–Mn–Ti and Zn–Cu–Ti alloys exhibited acceptable *in vitro* mechanical properties, appropriate degradation behaviors and *in vivo* safety and feasibility, thus were promising materials for clinical staples [69]. For orthopedic fixation devices, it is crucial to guarantee the anatomic reduction and stabilization of bone fractures while ensuring device degradability to avoid the need for removal procedures. Zn alloys, with their enhanced mechanical strength, appropriate degradation behavior, and good biocompatibility, are considered promising materials for such applications; by contrast, the fast degradation makes Mg alloys risky for load-bearing devices. Wang et al. evaluated the efficacy, biocompatibility, and degradation behavior of a Zn–Mg–Fe alloy internal fixation system applied to treat mandibular fractures in canines over 24 weeks, as represented in Fig. 2. The alloy had adequate mechanical properties that facilitated fracture healing. Zn alloy plates and screws corroded slowly and uniformly and maintained a complete shape, indicating their efficacy for fracture fixing (more than 24 weeks). Furthermore, the alloy showed good biocompatibility and was found promotive to new bone formation [70].

Batteries are key components for biodegradable electronic medicine, such as bioresorbable cardiac pacemakers. Primary biodegradable batteries based on Mg anodes, such as Mg–Mo and Mg–Fe, have been developed. However, Mg anodes often undergo intense side reactions with aqueous electrolytes due to their bioactivity, which not only limits their operational lifetime but also results in extra hydrogen production. Zn is considered as a promising alternative anode material for biodegradable batteries due to its fair biocompatibility, appropriate degradation behavior, high theoretical volume specific capacity, low equilibrium potential and high hydrogen evolution potential. Huang et al. successfully developed a fully biodegradable primary Zn–Mo battery, which had a prolonged operational lifetime up to 19 days, alongside desirable output voltage and energy capacity, as shown in Fig. 2. Additionally, Zn–Mo batteries showed outstanding biocompatibility and biodegradability both *in vitro* and *in vivo*, and promoted Schwann cell proliferation and the axonal growth of dorsal root ganglia. Remarkably, after being implanted in the subcutaneous area of the posterior back of SD rats for 12 weeks, the battery completely degraded [71].

GBR is an effective method for bone tissue augmentation to address the lack of bone volume around dental implants. An ideal GBR membrane must fulfill specific design criteria, including maintenance of space-making ability, excellent biocompatibility, proper degradability, adequate cell-occlusiveness, potential antibacterial activity, and clinical feasibility [72]. Zn-based BMs are promising materials for GBR membranes. They offer superior spacing capacity compared to polymer and collagen membranes, and a more stable degradation cycle than Mg-based BMs. As shown in Fig. 2, Guo et al. designed three kinds of pure Zn membranes, pure Zn without pores, pure Zn with 300 and 1000 μm diameter pores, and studied their *in vitro* and *in vivo* performance. Results showed that pure Zn membrane with 300 μm diameter pores exhibited the best osteogenic capability which was comparable to that of Ti membranes without pores. Sufficient mechanical properties and suitable degradable behavior were obtained except for the membrane with 1000 μm diameter pores. The study proved the feasibility of Zn-based BMs as GBR membrane materials [73]. In order to improve the overall performance of GBR membranes, Zhang et al. developed Zn-0.5Cu-xFe alloys. The Zn-0.5Cu-0.2Fe alloy demonstrated a notable UTS of 202.3 MPa and an elongation of 41.2 %, achieving a balance between strength and ductility. Furthermore, the alloy exhibited improved degradation behavior and good cytocompatibility, along with antibacterial properties, indicating its great potential in the field of GBR membranes [72].

Zn exhibits favorable biocompatibility within blood vessels and an appropriate rate of degradation, and has been extensively investigated as a material for cardiovascular stents [39,56]. In 2013, Bowen et al. first reported that Zn was a promising candidate for cardiac stents. Pure Zn wires were placed in the abdominal aorta of adult male SD rats for 6 months. Results showed that pure Zn had ideal physiological corrosion behavior, combined *in vivo* longevity with the harmless degradation [74]. However, the mechanical strength of pure Zn was insufficient for stent application. In recent years, various Zn alloys of superior strength have been developed [39]. For instance, Zhou et al. fabricated a novel Zn-0.8Cu coronary stent and implanted it into porcine coronary arteries for two years. This stent provided sufficient support in the initial stage of post-implantation. Additionally, the Zn–Cu stent displayed an appropriate degradation rate without accumulation of degradation products, thrombosis, or inflammatory response during *in vivo* tests [75]. In 2023, Yang et al. developed an ultrathin-strut (65 μm) Zn-0.1Li vascular scaffold, as illustrated in Fig. 2, aiming to decrease incidences of in-stent restenosis and thrombosis. By adding Li, the Zn-0.1Li scaffolds exhibited twice the radial force, while only having 40 % of the strut thickness compared to the pure Zn scaffolds. Additionally, the alloy scaffold demonstrated an impressive performance in preventing intimal hyperplasia and facilitating endothelialization [76].

As discussed in Sections 2.1, 2.4, Zn plays a vital role in stimulating bone formation and mineralization. Moreover, Zn-based BMs have a moderate degradation rate, and their mechanical strength and biocompatibility can be significantly improved by composition optimization. Therefore, Zn-based BMs have emerged as promising materials for orthopedic implants [36]. Li et al. evaluated the *in vivo* behavior of Zn–1Mg, Zn–1Ca and Zn–1Sr, which were implanted into mouse femora. No inflammation was observed around the implantation site. Zn alloys degraded slowly and the formation of gas shadow was not observed. Compared with the sham control group, the bone volume in Zn alloy groups was higher. Zn–1Sr showed the best osteogenic ability among all alloys [32]. Yang et al. utilized a rat femur condyle model to evaluate the *in vivo* behavior of pure Zn and Zn-HA, as shown in Fig. 2. Newly formed bone surrounding pure Zn and Zn-5HA was found at week 4 after implantation. The bone mass increased over time for both groups [36].

In addition to bulk materials, porous Zn-based BM scaffolds are also noteworthy for tissue-engineered bone implants, which require not only interconnected micropores similar to bone tissue for bone cell adhesion, growth, and blood vessel remodeling, but also a geometry that matches the bone defect for reliable fixation and stress distribution. Porous structure can also significantly reduce the elastic modulus of the implants, solving the urgent problem of high modulus of bulk Zn-based BMs mentioned in Section 2.3, thus decreasing the risk of stress shielding [9]. Additionally, porous designs can accelerate implant degradation, mitigating the issue of bulk Zn-based BMs remaining in the body longer than the bone healing period [15]. When it comes to the fabrication of scaffolds with such complex macro shapes and micro pores, conventional manufacturing techniques like casting and forging encounter difficulties. The pressure infiltration method has been used to manufacture metal porous scaffolds. However, this method has limited applications as it cannot accurately control the shape, and size of pores, nor can it facilitate the fabrication of devices with customized structures [77].

**Fig. 3:**
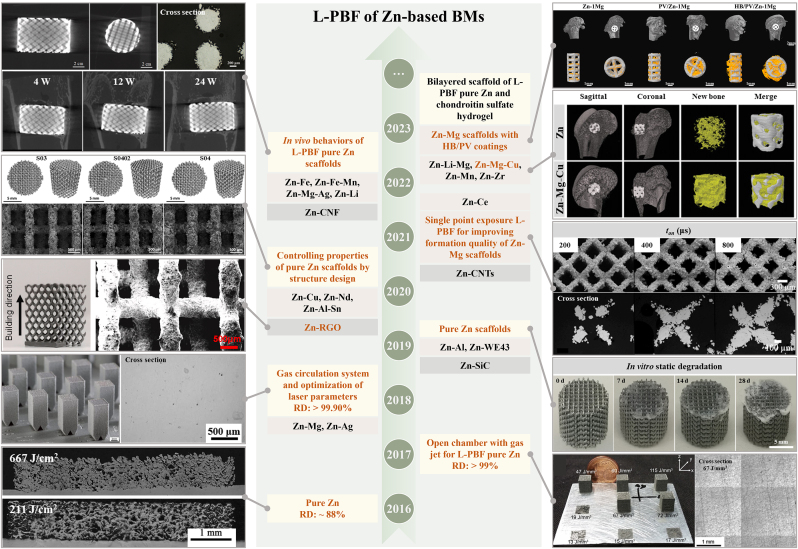
Timeline of research progress on L-PBF Zn-based BMs (Adapted from Refs. [12,18,[20], [21], [22], [23],^78^,^80^,^83^,^84^]).

L-PBF provides a solution for the fabrication of porous Zn-based BM implants of reliable properties, as shown in Fig. 3. In recent years, porous pure Zn scaffolds with designed structures have been successfully fabricated using L-PBF [21,88]. Meanwhile, in order to improve the comprehensive performance of L-PBF Zn-based BMs, the fabrication of Zn alloys and Zn-based composites has been extensively reported [14,18].

## 3 Zn-based BMs used in L-PBF

### 3.1 Pure Zn

Bulk pure Zn was first fabricated by L-PBF in 2016 (Fig. 3). However, the as-built block showed low densification with a RD of 88 % [12]. During L-PBF, pure Zn has a high evaporation tendency, due to its low melting and boiling points. The evaporation fume, containing small particles, attenuates the laser beam, leading to the unstable energy deposited on the powder bed, resulting in low densification. In order to improve the fusion quality, Demir et al. adopted an open chamber with an inert gas jet flow to blow off the evaporation fume. When the laser energy was in the range of 40–115 J/mm^3^, the RD of the bulk Zn exceeded 98 %, with the highest RD reaching approximately 99 % [78]. In 2018, Wen et al. developed a customized gas circulation system, which kept the oxygen content in the closed chamber below 50 ppm, while effectively purging metal vapor. In the processing window, the RD of the pure Zn was greater than 99.5 %. The hardness, E, TYS, UTS and elongation of pure Zn were 42 HV, 23 GPa, 114 MPa, 134 MPa and 10.1 % respectively, surpassing those of pure Zn fabricated by most traditional methods [20,79].

Porous pure Zn scaffolds were manufactured using L-PBF in 2019 by Li et al. The CYS and E of the scaffold with diamond cells and a porosity of 67 % were 10.8 and 786 MPa. After 4 weeks of dynamic and static immersion *in vitro*, the weight loss of scaffolds was 7.8 % and 3.6 %, respectively. Pure Zn scaffolds showed good biocompatibility *in vitro* [80]. To fully exploit the advantages of topological design of AM, Li et al. fabricated pure Zn scaffolds with functional gradient (diamond structure, unit size: 1.4 mm, S0402: struct thickness linearly and radially varied from 0.4 to 0.2 mm) and two uniform scaffolds (S03 and S04: struct thickness: 0.3 and 0.4 mm) to study their comprehensive performance. Their investigation revealed that structure design significantly influenced the scaffold's degradation behavior, leading to a 150 % variation in degradation rate between the three different structures. The CYS of all as-built pure Zn scaffolds was in the range of 4–11 MPa, and increased after the *in vitro* immersion [21]. Xia et al. implanted the pure Zn scaffold fabricated by L-PBF into the femur of rabbits and studied its osteogenic performance *in vivo*. The findings suggested that pure Zn scaffolds exhibited good osteogenic promotion ability, offering a promising solution for the treatment of large bone defects [22]. Yang et al. fabricated a biodegradable bilayered scaffold of chondroitin sulfate hydrogel and pure Zn scaffolds. The scaffold showed favorable biocompatibility and promoted chondrogenic and osteogenic differentiation. *In vivo* experiments demonstrated the potential of this fully biodegradable bilayer scaffold as a promising treatment for the repair of osteochondral defects, attributed to its biomechanical and bioactive gradients [81].

### 3.2 Zn alloys

In the pursuit of implants with superior comprehensive properties, various Zn alloys have been successfully manufactured using L-PBF. Yang et al. achieved the fabrication of Zn-xMg alloys (x = 0–4 wt%) by L-PBF. With 3 wt% Mg addition, the UTS and elongation were increased by 361 % and 423 % respectively compared with pure Zn. Moreover, the addition of Mg improved the corrosion resistance of Zn, the degradation rate decreased from 0.18 to 0.10 mm/year. The cell viability obtained from CCK-8 assay of Zn–3Mg on day 5 was 123.2 ± 4.2 %, higher than 91.3 ± 3.6 % of pure Zn, indicating the improved biocompatibility of Zn–Mg alloy [82]. Guaglione et al. developed a SPE strategy to suppress vapor and spark generation during L-PBF of Zn-based BMs. Zn-0.5Mg scaffolds with strut diameters smaller than 200 μm could be successfully fabricated [83]. Zhang et al. prepared HA/PDA composite coating, loaded with a bioactive factor BMP2 and an antibacterial drug Van, on L-PBF Zn–1Mg scaffolds by a molecular self-assembly process. The osteogenic and antibacterial activity of coated scaffolds was greatly enhanced [84]. In addition to Zn–Mg, other binary alloys, such as Zn–Li, Zn–Cu and Zn–Ce, have also been manufactured by L-PBF. The UTS of the Zn-0.7Li alloy reached 416.5 MPa [15]. The UTS of L-PBF Zn–3Cu was 223 MPa. Compared with pure Zn, Zn–Cu alloys exhibited greatly enhanced antibacterial activity. In addition, it also had favorable biocompatibility and suitable degradation rate [85]. The UTS of the L-PBF Zn–2Ce alloy was enhanced to 247.4 MPa, and the Zn–Ce implant exhibited strong antibacterial activity, good cytocompatibility, and hemocompatibility [16].

Ternary Zn alloys, like Zn-Mg-Ag [86], Zn-Fe-Mn [87], Zn-Mg-Cu [23] and Zn–Li–Mg alloys [88], have been successfully fabricated by L-PBF to further improve the performance of binary alloys. Zn–3Mg–1Ag alloy had a high CS of 309 MPa and an improved strain of 27 %. Moreover, the alloy also exhibited high biocompatibility and strong antibacterial activity [86]. Zn–2Fe–2Mn alloy exhibited a UTS and elongation of 226.8 MPa and 15.9 %, which were 25 %, and 87 % higher than those of Zn–2Fe alloy [87]. L-PBF Zn–3Mg–2Cu porous scaffolds with gyroid structure showed enhanced osteogenesis, immunoregulation, angiogenesis and anti-infective activity [23]. L-PBF bulk Zn-0.8Li-0.1Mg had a UTS of 460.78 MPa, which was more than 3 times that of pure Zn. Compared with pure Zn, the alloy had a higher corrosion resistance, better biocompatibility and osteogenic ability [88].

### 3.3 Zn-based composites

In addition to Zn alloys, researchers have also fabricated Zn-based composites exhibiting enhanced performance by L-PBF. Reinforcing materials such as nano-SiC, RGO, CNTs, and CNF have been employed in these composites. The CYS (121.8 MPa) of Zn–2SiC was increased by 441 % in comparison to pure Zn [17]. To address the challenge of weak interface bonding between nano-SiC and the Zn matrix, Gao et al. employed a pre-oxidation technique for the nano-SiC and Zn powders. This method facilitated an in-situ reaction that enhanced interface bonding, resulting in a 31.6 % increase in CYS of materials [89]. Yang et al. realized the fabrication of Zn-RGO using L-PBF. The UTS of Zn-0.2RGO increased from 119.9 MPa to 182.1 MPa, and the elongation improved from 9.5 % to 14.1 % compared with pure Zn. Furthermore, Zn-RGO was found to promote cell growth and differentiation [18]. Additionally, TiC was utilized as an interfacial bridge to improve the bonding between RGO and the Zn matrix. The UTS of the Zn–TiC@RGO scaffold was increased from 45.9 MPa of Zn-RGO to 59.9 MPa [90].

The Zn-CNTs composite exhibited a UTS of 175 MPa and an elongation of 13.8 %, surpassing the 98 MPa and 7.4 % of pure Zn. The strong agglomeration and weak interface boning between CNTs and Zn matrix restricted the reinforcing effect. To address this, Ag nanoparticles were introduced. Compared with Zn-CNTs, the UTS and elongation of Zn-CNT@Ag increased by 26 % and 17 %, respectively. This composite demonstrated good antibacterial acativity, suitable corrosion rate of 0.21 mm/year, and acceptable biocompatibility [19]. Yang et al. fabricated Zn–CNF by L-PBF and used La as the compatible interface layer between CNF and Zn matrix to reinforce the interfacial bonding. The UTS and elongation of Zn–La@CNF were improved to 243.4 MPa and 9.3 % from 180.2 MPa and 8.1 % of Zn–CNF. Furthermore, Zn–La@CNF exhibited remarkable anti-tumor efficiency and the addition of La@CNT showed no adverse impact on the growth and differentiation of normal cells [91].

## 4 Key issues on Zn-based BMs fabricated by L-PBF

### 4.1 Powder particles

**Fig. 4:**
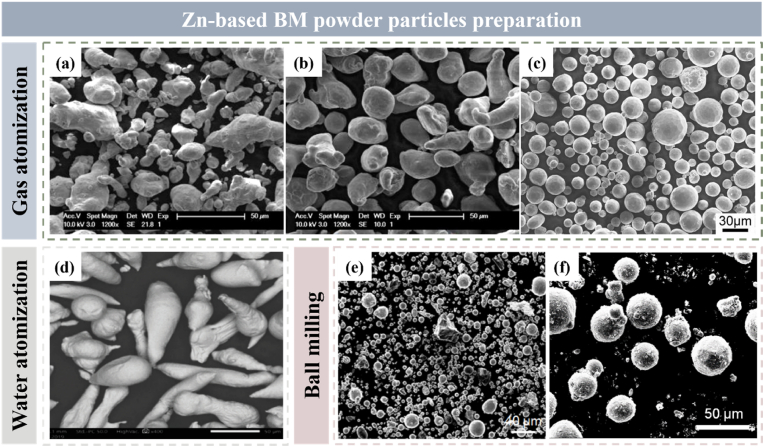
Morphologies of powder particles: Pure Zn: (a) prepared by air atomization [94]; (b) and (c) prepared by nitrogen atomization preparation [20,94]; (d) prepared by WA [95]; (e) Zn-Fe-Mn [87] and (f) Zn-RGO powders [18] by ball-milling.

Powder particles used for L-PBF should meet the requirements of high purity, high relative density, good sphericity, uniform composition distribution and uniform particle size distribution [9,92]. High purity guarantees the desired composition of the fabricated components, while a high RD, indicating fewer gas pores in powders, is crucial for achieving a high fusion quality. The shape and size distributions of powders affect their flowability, which exerts a considerable impact on powder spreading, and consequently influences the fusion quality of the fabricated material [11,93]. Currently, the preparation of Zn-based BM powders usually adopts GA and WA methods. Lietaert et al. prepared pure Zn powders using air atomization and nitrogen atomization. As shown in Fig. 4a–b, compared with the powder atomized by air, the powder atomized by nitrogen had higher sphericity, indicating better fluidity. The powder atomized by air contained more small particles. The results showed that the bulk materials fabricated by powders obtained by nitrogen atomization have better fusion quality, with a RD higher than 99.70 %, compared with those produced by air atomized powders [94]. Ruvalcaba et al. obtained pure Zn powder by WA, and the morphology of powders was shown in Fig. 4d. The powder had poor sphericity, irregular shape and poor fluidity. The maximum RD of as-built bulk samples was only 95–97 % [95]. Demir et al. used Zn powders with average sizes of 15 μm and 9 μm, prepared by WA, to fabricate bulk materials. They discovered that larger powder sizes could achieve superior fusion quality, with the RD exceeding 99 % [78]. Wen et al. manufactured pure Zn using nitrogen atomized powders, exhibiting a spherical morphology as displayed in Fig. 4c. The as-built materials had a RD surpassing 99.5 % [20].

In addition to the morphology, the chemical composition of alloy powders by GA and WA methods is rarely noticed but equally important. Due to the melting process of the ingots in atomization and the varying evaporation tendencies of different elements, the chemical composition of powders often diverges from the originally designed composition. For instance, the Li content of Zn–Li ingots was 0.8 wt%. While, after the GA, the Li content of powders was only 0.69 wt% [15]. Similarly, the Mg content in Zn–5Mg alloy reached 5.035 wt%, exceeding the targeted 5 wt% in ingots [14]. Therefore, it is of great importance to consider the change of composition in advance when preparing alloy powders by atomization methods. To obtain Zn alloy and Zn-based composite powders, mechanical mixing methods, such as ball-milling, are also employed. Fig. 4e and f showed the morphology of Zn–Fe–Mn and Zn-RGO mixed powders [18,87]. The change in chemical composition of powders is not a concern for mechanical methods because there is no melting stage in preparation processes. However, they can lead to compositional segregation and bad fusion quality due to the powders with inhomogeneous composition distribution and poor sphericity [11,96].

### 4.2 Structure design

**Fig. 5:**
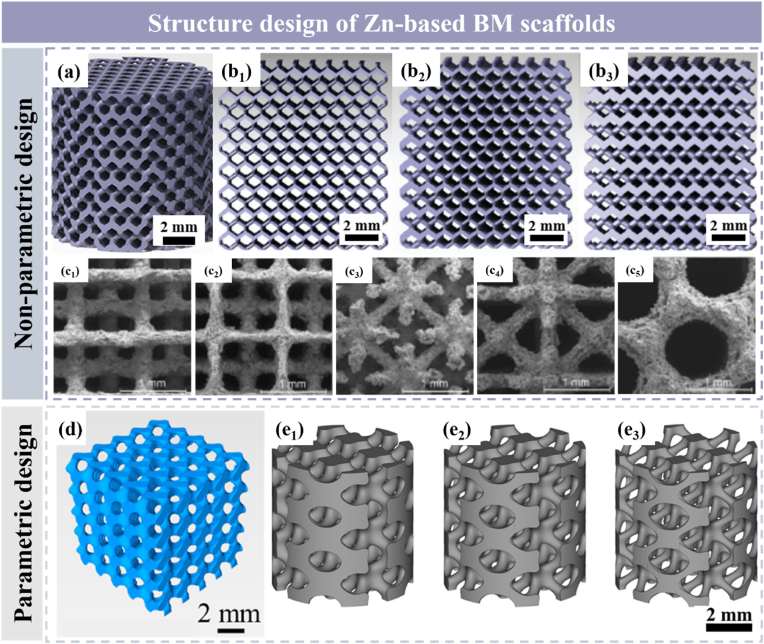
Structure design: (a) Schematic diagram of the scaffolds [80] (diamond structure, porosity: 67 %); (b) schematic diagram of porous scaffolds with structural gradients [21]: (b_1_) S03 (porosity: 81.1 %), (b_2_) S0402 (porosity: 74.3 %), (b_3_) S04 (porosity: 67.4 %); (c) morphology of porous scaffolds with different units [98]: (c_1_) Diamond unit, (c_2_) Dodecahedron unit, (c_3_) Octet truss unit, (c_4_) FCC unit, (c_5_) 3D Kagome unit, (d) TPMS gyroid structure [104], (e) TPMS IWP structures with different porosities: 60 % (e_1_), 70 % (e_2_), and 80 % (e_3_) [88].

Porous implants, comprising multiple unit cells, have their performance significantly influenced by the characteristics of these unit cells, such as unit type, porosity, and unit size. Consequently, the unit cell should be carefully selected when designing the structure of scaffold. Unit cells are mainly generated by two methods, non-parametric and parametric designs [97]. Non-parametric design, also referred to geometry-based design, is predominantly generated using CAD methods. A typical example of a non-parametric cell is the diamond cell, extensively utilized in bone implants. The first L-PBF pure Zn scaffolds had the unit type of diamond with a porosity of 67 %, as shown in Fig. 5a [80]. Li et al. further designed and manufactured pure Zn scaffolds with diamond units of different porosity distributions (Fig. 5b) [21]. In addition to diamond unit, non-parametric unit cells, such as dodecahedron, octet truss, FCC and 3D Kagome, were also utilized for the fabrication of pure Zn scaffolds (Fig. 5c) [98].

Although the non-parametric design is simple and widely studied, the unit cells contain sharp transitions between struts, where stress concentrations occur, making them less suitable for orthopedic applications [99]. Parametric design, which utilizes algorithms to generate cells, provides a way for the optimization of structure design. Among all parametric design methods, TPMS is most widely reported in the field of bone implants. Unit cells based on TPMS have advantages of high permeability, smooth transitions and excellent pore connectivity. The stress contribution of scaffolds is more uniform under compression due to the smooth transitions. Moreover, except for the optimized mechanical behaviors, TPMS units are also able to mimic biological bone tissue to integrate well with surrounding tissues and facilitate cellular processes, like oxygen diffusion, ion exchange and nutrient transport [[100], [101], [102], [103]]. Therefore, TPMS units like the gyroid and IWP, have been utilized in the design and fabrication of Zn-based BM scaffolds for orthopedic implants. Zhao et al. fabricated Zn alloy scaffolds with gyroid units, as shown in Fig. 5d. The TPMS structure provided high relative surface areas and improved permeability, promoting cell adherence and retention. Furthermore, cell-level directional curvatures of gyroid units elongated the cells’ morphology and strengthened cytoskeletal contraction, thus obtaining better osteogenic outcomes [23,104]. Liu et al. fabricated IWP unit-based Zn-0.8Li-0.1 Mg scaffolds with porosities of 60 %, 70 % and 80 %, as shown in Fig. 5e [88].

### 4.3 Processing optimization

#### 4.3.1 Fusion quality

**Fig. 6:**
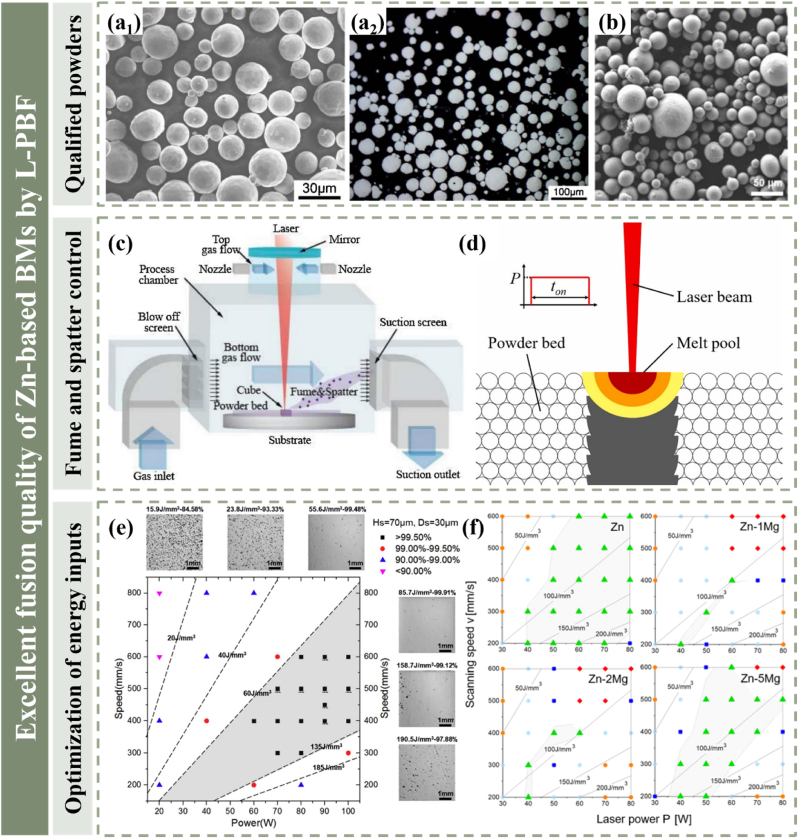
Three main factors for improvement of fusion quality of Zn-based BMs by L-PBF: qualified powders: (a_1_) powder shape, (a_2_) powder cross section of pure Zn powders [79], (b) Zn-0.7Li powders [15]; fume and spatter control: (c) gas circulation system [79], (d) schematic figure of the single pulse interaction (P: peak laser power, *t*_on_: certain duration) [83]; optimization of energy input: processing windows of (e) pure Zn [20], (f) Zn-xMg alloys [115].

Poor fusion quality, majorly reflected by the occurrence of lack of fusion and porosity, can greatly deteriorate comprehensive properties of additively manufactured components [109]. The RD of the first reported L-PBF pure Zn was only 88 % [12]. To achieve optimized fusion quality, it is essential to focus on three primary factors: the characteristics of the powder particles, the control of fume and spatter, and the careful selection of processing parameters, as illustrated in Fig. 6. The preparation and quality control of powder particles, which are critical for enhanced fusion quality, have been elaborated in Section 4.1. Concerning fume and spatter control, the low boiling point of Zn contributes to the generation of substantial evaporation fumes during L-PBF. Demir et al. used infrared cameras to capture the behavior of fume and spatter in the process of L-PBF of Zn. The scattering of laser beams by particles in the fume made the laser energy focused on the powder bed unstable, and eventually led to poor fusion quality of the material [[110], [111], [112]]. To improve the fusion quality, Demir et al. adopted an auxiliary purge gas system, in which inert gas flowed over the powder bed in an open chamber to blow away metal vapors and particles. The highest RD of the bulk Zn was over 98 % [78]. Wen et al. developed a closed process chamber with a customized gas circulation system, as shown in Fig. 6c. Due to the good control of fume and spatter by utilizing auxiliary inert gas in the chamber with extremely low content of oxygen, the RD of L-PBF bulk Zn exceeded 99.5 % [79]. In addition to controlling smoke by blowing it away, strategies aimed directly at inhibiting excessive smoke formation have proven to be effective. Guaglione et al. created the SPE scanning strategy to suppress excess formation of vapor during the L-PBF of Zn–Mg alloy scaffolds. Unlike conventional linear vector scanning, the SPE process utilized single laser pulses at specific locations to optimize the energy input to melt the material while avoiding the formation of metal vapors, improving manufacturing stability. The schematic figure of the single pulse interaction was shown in Fig. 6d. The results showed that there was almost no smoke and spark formation in the L-PBF process, and the printed scaffolds showed good fusion quality [83].

To achieve excellent fusion quality of Zn-based BMs, precise control of laser energy input is essential. In L-PBF, laser power (*P*), scanning speed (*v*), hatching space (*Hs*) and layer thickness (*Ds*) significantly impact the fusion quality. The specific laser energy density (*Ev*), as defined in Eq. (7), is employed to represent laser energy input. An inadequate *Ev* results in insufficient powder melting, leading to a lack of fusion defect. In contrast, if *Ev* is too high, evaporation will lead to local accumulation of vapor pressure inside the molten pool, and the molten material will spatter and produce keyhole defects. Therefore, high fusion quality can be obtained only by selecting appropriate laser energy input [8,113,114]. Wen et al. investigated the processing window of pure Zn by varying the laser power and scanning speed, as shown in Fig. 6e. Based on varying energy densities, the processing window can be classified into six distinct areas. In the upper left corner, the energy input was the lowest. Lack of fusion defects with irregular shapes occurred between layers and tracks, and the RD was less than 90 %. In the lower right corner, the energy input was too high, and excessive evaporation led to the formation of circular pores. When the *Ev* was in the range of 60–135 J/mm^3^, the relative density could exceed 99.5 %. Owing to the characteristics of the keyhole, pores in the material cannot be eliminated, thereby the perfect fusion quality with a relative density of 100 % cannot be achieved [20].

(7) $$E_{v}=\frac{P}{v\times Hs\times Ds}$$

As previously noted, the processing window for pure Zn has been established. Regarding the processing windows of Zn alloys, some studies on Zn–Mg and Zn–Li have been published. Notably, even minor variations in chemical composition significantly modify the processing windows of Zn alloys. Compared to pure Zn, the processing windows for these alloys are notably narrower. Voshage et al. explored the processing window of Zn-xMg, as shown in Fig. 6f. The optimized *Ev* of pure Zn, Zn–1Mg, Zn–2Mg and Zn–5Mg was 70–230, 100–150, 75–125 and 75–150 J/mm^3^, respectively. Among these Zn–Mg alloys, Zn–5Mg had the largest processing window. The processing window of the material was negatively correlated with the depth of the molten pool, and the depth was correlated with the melting temperature of the material. Compared with the other two Zn–Mg alloys, Zn–5Mg had a higher melting temperature and lower melting depth, thus it had a larger processing window [14,115]. Qin et al. obtained the processing window of Zn-0.7Li alloy. Only one *Ev*, 36 J/mm^3^, could manufacture the bulk components with a RD greater than 99.5 %, attributed to the addition of Li [15].

#### 4.3.2 Dimensional accuracy

L-PBF is employed for the fabrication of components with customized shapes and complex structures. For the formation quality, the dimensional accuracy of fabricated components, reflected by geometric error between the design and the fabricated structure, is equally significant. This accuracy is a key measure of the effectiveness of L-PBF in producing designs as intended. The dimensional accuracy can be controlled by adjusting the energy input in L-PBF. Qin et al. studied the relationship between the dimensional accuracy of Zn-0.7Li scaffolds and the energy density. At the maximum *Ev* (*P* = 120 W, *v* = 200 mm/s), the particles attached to the scaffold surface seriously blocked the pore structure, and the porosity of the scaffold was only 56.34 %, much lower than the design value of 80 %. When the *Ev* was minimum (*P* = 40 W, *v* = 1000 mm/s), the dimensional accuracy of the scaffold was the highest with a porosity of 78.88 %. Considering the fabricated scaffolds should have high dimensional accuracy on the premise of high fusion quality, *P* = 40 W and *v* = 1000 mm/s was selected as the optimal processing parameters for scaffold preparation [15]. Fortunately, the simultaneous attainment of satisfactory fusion quality and dimensional accuracy is possible for Zn–Li alloy scaffolds. This is due to the notably low optimized energy input required for high fusion quality in Zn–Li alloys, which is advantageous for ensuring dimensional accuracy. However, in the case of some Zn-based BMs, achieving both high fusion quality and dimensional accuracy concurrently is not feasible. Qin et al. fabricated Zn–Mg alloy scaffolds by L-PBF. By employing parameters optimized for high fusion quality, the actual porosity of scaffolds was only 50.4–52.8 %, while the designed porosity was 66.7 % [14]. Additionally, Zn-WE43 alloy scaffolds demonstrated suboptimal dimensional accuracy, with porosity deviating by 22 % from the designed value [116].

To date, no systematic research has been conducted to investigate the poor dimensional accuracy of Zn-based BM scaffolds. While, this phenomenon can potentially be explained by theories from Mg alloys, which share similar physical and chemical properties with Zn. Wen et al. fabricated WE43 Mg alloy scaffolds and studied the reasons for the poor dimensional accuracy of scaffolds. The relative density of WE43 scaffold exceeded 99.5 % when *P* = 60 W and *v* = 600 mm/s, but the dimensional error of scaffolds fabricated using such heat input was 33.8–96.8 %. Mg had a high thermal expansion coefficient and low surface tension, resulting in molten materials spreading out perpendicular to the BD. Furthermore, the low melting point and high chemical reactivity of Mg alloys result in powders easily adhering to the struts, diminishing accuracy. Finally, similar to Zn, due to the low boiling point of Mg, violent evaporation occurred during the L-PBF process, generating an outward laser plume perpendicular to BD. The ejection of the laser plume created a low-pressure area, and the gas flowed inwards and carried the surrounding powders into the molten pool, causing more powder melting and increasing the volume of the scaffold [117]. In order to improve the dimensional accuracy of Zn-based BM scaffolds, various surface treatment methods including sandblasting, chemical polishing, and electrochemical polishing have been employed and proven effective [118].

### 4.4 Chemical compositions

The chemical composition significantly influences the properties of Zn-based BMs. Jin et al. reported that the UTS of hot-extruded Zn–Mg alloy rose markedly from 63 MPa to 202 MPa with a slight increase in Mg content from 0.002 wt% to 0.005 wt% [119]. Thus, considering the change in chemical composition is crucial when manufacturing Zn-based BMs. In L-PBF, the laser has extremely high energy density, leading to high molten pool temperature, thus causing the burning loss of elements. When fabricating alloys, elements evaporate and burn, while the evaporation rate of different elements is different. As a consequence, the chemical composition of as-built materials deviates from that of powders [120,121]. Qin et al. reported the chemical composition of Zn–Mg alloy by L-PBF. The Mg content of as-built Zn–1Mg, Zn–2Mg and Zn–5Mg alloys was 1.337 %, 2.244 % and 5.206 %, higher than 1.003 %, 2.034 % and 5.035 % of powders. Compared with Mg, Zn had a lower boiling point and a higher saturated vapor pressure, thus the evaporation of Zn was greater than Mg in L-PBF, leading to the increase of Mg element content in fabricated parts [14]. For Zn-0.7Li alloy, the content of Li in L-PBF samples decreased from 0.690 wt% of powders to 0.675 wt%. The vaporization flux of elements in an alloy was positively related to its equilibrium vapor pressure and negatively related to its molecular weight. Although the equilibrium vapor pressure of Li was lower than that of Zn, Li had an extremely low molecular weight, so it evaporated more [15]. In L-PBF, changes in chemical composition are inevitable due to the varying vaporization tendencies of different elements. Therefore, it is crucial to consider these compositional changes when designing the element content of powders, to ensure that the as-built samples have the intended composition.

### 4.5 Surface modification

The interaction between implants and tissues is significantly influenced by the surface properties of the implants. During degradation, Zn-based BMs release Zn^2+^, which is cytotoxic at high concentrations, and thus exhibit unsuitable biocompatibility with natural bone tissue. Surface modification emerges as a highly effective method to improve surface properties and introduce new functions to implants. This technique only alters the surface characteristics of Zn-based BMs while maintaining their original performance [105,106]. There has been an increasing number of studies on the surface modification of bulk Zn-based BMs in recent years. Various techniques, such as ALD and biomimetic conversion, have been employed to modulate degradation rates and improve biocompatibility for orthopedic applications. Yuan et al. applied ALD to deposit a homogeneous ZrO_2_ film on Zn–Li alloy surfaces. This modification significantly enhanced the corrosion resistance of the alloy and reduced Zn^2+^ release. It also facilitated better cell adhesion, proliferation, and osseointegration [107]. Jablonská et al. treated Zn-1.5Mg alloy with simulated body fluid. A protective layer, abundant in calcium phosphate was formed on the surface, leading to enhanced corrosion resistance and improved cell viability and adhesion [108].

**Fig. 7:**
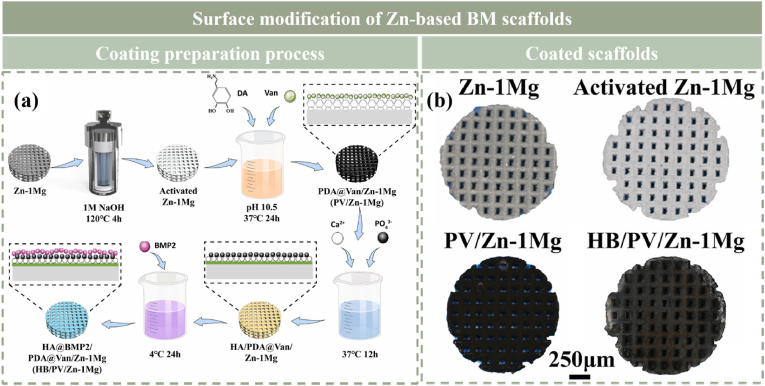
Surface modification of Zn-based BM scaffolds: (a) Schematic diagram of preparation process, (b) Overview pictures of coated samples [84].

Surface modification of Zn-based BM scaffolds is an emerging field, presenting unique challenges in achieving uniform and dense coatings on their intricate internal porous structures. Zhang et al. first realized the preparation of HA/PDA composite coating, loaded with a bioactive factor BMP2 and an antibacterial drug Van, on L-PBF Zn–1Mg scaffolds by a molecular self-assembly process, as shown in Fig. 7. The specific characteristics and structures of L-PBF Zn-based BM scaffolds pose several obstacles to the preparation of coatings. Firstly, the hydrophobic nature of Zn surfaces hinders coating adherence. Secondly, the slight degradation of Zn-based BMs in the coating deposition solution disrupts the coating adhesion. Finally, the complex structure of porous scaffolds makes the uniform deposition of coatings on inner surface difficult. To address these issues, they prepared a uniform layer of ZnO on the surface of Zn–Mg scaffolds via alkali-heat treatment. This layer not only enhanced the surface wettability, facilitating coating adhesion, but also provided stable binding sites for PDA adherence, effectively overcoming the inherent challenges of scaffold surface modification [84].

### 4.6 Microstructure

**Fig. 8:**
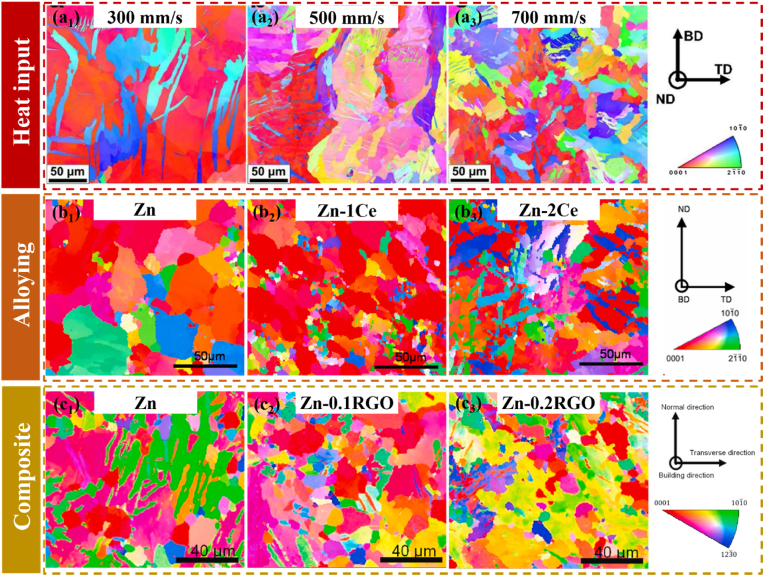
Microstructure of L-PBF Zn-based BMs: Inverse pole figures of L-PBF (a) pure Zn fabricated by different heat inputs (adapted from Ref. [124]), (b) Zn–Ce alloy (adapted from Ref. [16]), and (c) Zn-RGO composites (adapted from Ref. [18]).

Grain size in AM is mainly determined by the cooling rate during the solidification. The cooling rate of L-PBF is around 10^5^–10^6^ K/s. Such a high cooling rate results in the fine grain size, greatly improving the strength of the material [5,122]. For instance, although the relative density of L-PBF bulk Zn was only 88 %, its strength was still higher than that of Zn samples fabricated by traditional manufacturing methods, explained by fine grains of L-PBF parts [12]. Due to the improved mechanical strength from the fine grain strengthening, fine grains are desired to be obtained in Zn-based BMs. Three primary methods are commonly employed to adjust grain size in L-PBF. Firstly, the grain size can be controlled by heat input. The lower heat input is, the higher cooling rate is, indicating the finer grain size. Shuai et al. studied the relationship between grain size and heat input. Grain size increased with increasing heat input [123]. Qin et al. investigated the effect of scanning speed on the grain size of L-PBF pure Zn. Grain size gradually decreased with increasing scanning speed at a constant power. As shown in Fig. 8a, when the scanning speed was 300 mm/s, the coarse columnar grains were observed. When the scanning speed was increased to 700 mm/s, the coarse columnar grains changed to refined polygon grains, and the average grain width was only 5.9 μm. The higher scanning speed indicated the lower heat input. This reduced heat input results in less heat accumulation within the molten pool, thereby facilitating more efficient heat dissipation. Consequently, this led to an increased cooling rate, which was instrumental in obtaining finer grain sizes [124,125].

Secondly, alloying has been proven to be an effective method for controlling grain size [51,126]. Shuai et al. employed Ce, Cu, and Ag as alloying elements, successfully achieving grain refinement. When 2 wt% of Ce was added to Zn, as shown in Fig. 8b, the grain size decreased from 8.7 μm of pure Zn to 3.9 μm. Ce had high surface activity, leading to the formation of a surfactant film, which could reduce the surface tension of the liquid phase, thus reducing the nucleation energy and critical nucleation radius for heterogeneous nucleation. At the same time, the CeZn_3_ phase with high melting point precipitated preferentially during the solidification process, leading to component fluctuation and undercooling at the solid/liquid interface, thus limiting the growth of primary grains [16]. In the case of Zn–Cu alloys, the grain sizes of Zn–1Cu, Zn–2Cu, and Zn–3Cu were measured at 24.3, 15.6, and 9.8 μm, respectively, all significantly smaller than the 56.7 μm of pure Zn. However, further increases in Cu content to 4 wt% did not result in further grain size refinement. When Cu content was lower than its maximum solubility, 1.7 wt%, in Zn, no CuZn_5_ phase precipitated in the microstructure. Cu produced constitutional supercooling at the solid/liquid interface, providing the driving force for nucleation, restricting the growth of primary grain. When the Cu content exceeds 2 wt%, CuZn_5_ phase precipitated in the microstructure, providing a heterogeneous nucleation site for Zn, thereby refining the grain size [85]. Regarding Zn–Ag alloys, a gradual decrease in grain size was observed with increasing Ag content, reaching its minimum at 6 wt% Ag. However, further increasing Ag content to 8 wt% did not continue to reduce grain size but instead resulted in an increased grain size. Ag could lead to component supercooling at the solid/liquid interface during solidification and promote nucleation. When the content of Ag was too high, the secondary phase AgZn_3_ grew rapidly and connected, eventually leading to an increase of grain size [127].

Finally, the doping of reinforcement materials, such as nano-SiC, RGO and CNF, has been shown to effectively refine grain size of Zn-based BMs. Gao et al. refined the grain by adding nano-SiC to pure Zn matrix. The grain size of L-PBF pure Zn was about 250 μm. After adding nano-SiC, the grain size of Zn-0.5SiC, Zn–1SiC and Zn–2SiC decreased to 90 μm, 50 μm and 15 μm, respectively. However, the grain size distribution became nonuniform when the SiC content further increased to 3 wt%, explained by the agglomeration or inhomogeneous distribution of excessive nanoparticles. During solidification, undissolved nanoparticles provided heterogeneous nucleation sites for Zn, thus restricting the growth of Zn grain [17]. Yang et al. fabricated Zn-RGO composites and reported the grain size distribution, as shown in Fig. 8c. Compared with the average grain size of 6.9 μm in pure Zn, the grain size of Zn-0.2RGO and Zn-0.3RGO decreased to 3.1 μm and 2.4 μm, respectively. Similar with the grain refinement mechanism of Ce, RGO also had high surface energy, thus reducing the nucleation energy and critical nucleus radius of heterogeneous nucleation. Moreover, RGO had high thermal conductivity, leading to the enhancement of undercooling at the solid/liquid interface, inducing the growth of fine equiaxed grains, therefore contributing to the reduction of grain size [18]. Regarding CNF, the addition of CNF to pure Zn led to a reduction in grain size from 12.3 μm to 6.7 μm, attributed to its restriction for the growth of primary grains [91].

In addition to grain size, the texture significantly influences the material's properties and should therefore be duly considered. As shown in Fig. 8a_1_, due to the great temperature gradient in the solidification process of L-PBF, the microstructure of the pure Zn is mainly composed of columnar grains, growing along the BD, with an orientation of <0001>. Strong texture can lead to anisotropy and is detrimental to the ductility of the material, therefore, it should be weakened during the manufacturing process [124,128,129]. Measures including altering scanning speed, doping reinforcements, and alloying have been demonstrated to effectively control texture. Qin et al. investigated the effect of scanning speed on the texture of pure Zn. When the scanning speed was 300 mm/s, there was a strong ND//<0001> texture and a BD//<10-10> texture. With the increase of scanning speed, the maximum intensity of pore figures decreased from 36.62 (300 mm/s) to 8.08 (700 mm/s). Increasing scanning speed increased the cooling rate, reduced the time for the growth of columnar grains, and resulted in the formation of more refined polygon grains, thereby weakening the texture [124]. Yang et al. achieved a reduction in the TI of L-PBF Zn BMs by adding the rare earth element Ce. The TI decreased from 19.93 in pure Zn to 7.71 in Zn–2Ce. Ce reduced the surface tension of liquid phase, leading to the reduction of nucleation energy and critical nucleation radius for heterogeneous nucleation. At the same time, the precipitation of secondary phase also hindered the growth of primary grains along the same orientation, so the texture was weakened [16]. Yang et al. weakened the texture by doping RGO. Pure Zn grains mainly grew into (0001) orientation, while, for Zn-RGO, the grain orientation along (0001) was weakened. The fiber TI of pure Zn was 20.5, while, the TI of Zn-0.2RGO was only 7.3. Due to the high thermal conductivity of RGO, the undercooling at the solid/liquid interface during solidification increased, providing low-energy-barrier heterogeneous nucleation sites, and inducing the nucleation of fine equiaxed grains. The newly nucleated grains had different orientations, thus weakening the texture [18].

### 4.7 Mechanical properties

The mechanical behaviors of Zn-based BM implants are contingent upon the material composition and structure design. As detailed in Table 1, various Zn alloys and Zn-based composites, exhibiting enhanced mechanical properties, have been successfully fabricated using L-PBF in recent years. Notably, Mg and Li have been identified as effective elements for enhancing strength. Zn–1Mg and Zn-0.7Li had a UTS of 381 and 416.5 MPa respectively. Zn-0.8Li-0.1 Mg alloy had a UTS of 460.78 MPa, which was the highest value among all reported L-PBF Zn alloys [14,15,88]. Alloying improves the strength mainly by grain refinement, second phase strengthening and solution strengthening, as mentioned in Section 2.3. Regarding Zn-based composites, doping nanoparticles significantly improve the mechanical strength by grain refinement strengthening, load transfer strengthening, Orowan strengthening and thermal mismatch strengthening [13,130]. As discussed in Section 4.6, the doping of nanoparticles achieves remarkable grain refinement, thus improving the strength of the material. As for load transfer strengthening, when the external force is applied to the material composited by nanoparticles, the load is transferred from the Zn matrix to the reinforcements through the interfacial shear stress. Massive fracture energy is consumed by reinforcements to prevent the crack propagation, realizing the improvement of strength [17,18,130]. Orowan strengthening refers to the phenomenon where uniformly distributed nanoparticles in the Zn matrix accumulate, pin down, and form dislocation loops, thus hindering the propagation of dislocation [90,131]. Finally, due to the large difference in thermal expansion coefficients between matrix and reinforcements, the thermal mismatch strengthening also improves the strength of materials [90].

**Table 1:** Fusion quality and mechanical properties of Zn-based BM bulk materials fabricated by L-PBF.

Composition (wt.%)	RD (%)	Mechanical properties^a^							References
		Hardness (HV)	TYS (MPa)	UTS (MPa)	E (%)	CYS (MPa)	CS (MPa)	E (GPa)	
**Pure Zn**									
Zn	88	/	/	/	/	99	/	/	[12]
	>99	42	/	/	/	/	/	/	[8]
	>99.9	46.31	122.13	137.9	8.13	/	/	20.47	[79]
	>99.5	42	114	134	10.1	/	/	23	[20]
	>99.5	/	110.3	132.9	∼7.5	/	/	/	[124]
	>98	/	78	100	10	/	/	88	[98]
	91.24–93.04	39.03	84.29	95.93	11.73	/	/	/	[140]
	>99.5	50.2	100.7	127.8	7.6	/	/	/	[141]
	97.4	49.5	43.2	61.3	1.7	/	/	12.2	[82]
	/	41.2	/	118	26.1	/	/	/	[14]
	97.4	37	/	/	/	/	145	/	[127]
	/	41	/	86	10.6	71	/	/	[85]
	90.53	56.5	/	66.5	10.2	/	/	58.4	[142]
	/	/	79.9	103.6	5.1	/	/	/	[16]
	/	35	/	/	/	62	/	/	[143]
	/	44.73	83.7	106.7	6.3	61.75	/	/	[87]
	/	41	/	/	/	22.4	/	/	[17]
	/	/	91.6	119.9	9.5	/	/	/	[18]
	/	45	76	98	7.4	85	/	4.7	[19]
	/	49.5	68.7	106.6	6.5	/	/	/	[91]
	/	48.16	102.18	134.02	42.20	/	/	57.90	[88]
**Zn alloys**									
Zn–1Mg	97.8	93.2	74.1	126.3	3.6	/	/	∼20	[82]
Zn–2Mg	96.6	138.4	117.4	161.8	4.1	/	/	∼25	[82]
Zn–3Mg	98.2	177.2	152.4	222.3	7.2	/	/	48.2	[82]
Zn–4Mg	98.1	198.6	131.6	166.4	3.1	/	/	57.5	[82]
Zn–1Mg	>99.5	114.8	/	381	4.2	/	/	∼80	[14]
Zn–2Mg	>99.5	150.1	/	283	<1	/	/	∼80	[14]
Zn–5Mg	>99.5	200.4	/	64	<1	/	/	∼80	[14]
Zn–10Mg	≥99.6	298	/	/	/		214	/	[144]
Zn-2WE43	99.93	114	/	298.5	1.8	/	/	/	[116]
Zn-5WE43	99.75	146	/	335.4	1	/	/	/	[116]
Zn-8WE43	99.47	169	/	154.1	0.9	/		/	[116]
Zn–2Ag	97.8	55	/	/	/	/	199	/	[127]
Zn–4Ag	98.2	80	/	/	/	/	216	/	[127]
Zn–6Ag	97.9	80	/	/	/	/	293	/	[127]
Zn–8Ag	98.5	∼80	/	/	/	/	267	/	[127]
Zn–2Al	98.3	64.5	141.7	192.2	11.7	/	/	/	[123]
Zn–1Cu	/	63	/	∼150	∼16	92	/	/	[85]
Zn–2Cu	/	78	/	∼180	∼19	118	/	/	[85]
Zn–3Cu	/	97	/	223	22.5	165	/	/	[85]
Zn–4Cu	/	90	/	207	∼21	156	/	/	[85]
Zn-1Nd	/	67.2	/	96.3	8.7	/	/	67.4	[142]
Zn-3Nd	98.71	76.2	/	119.3	6.7	/	/	79.4	[142]
Zn-5Nd	/	82.2	/	107.3	4.3	/	/	85.4	[142]
Zn–1Ce	/	/	∼140	∼200	∼6	//	/	/	[16]
Zn–2Ce	/	/	180.6	247.4	7.5	/	/	/	[16]
Zn–3Ce	/	/	∼190	∼220	∼6.8	/	/	/	[16]
Zn-0.7Li	>99.5	113.4	359.1	416.5	2.3	/	/	83.3	[15]
Zn–5Mn	/	∼50	/	/	/	∼140	/	/	[143]
Zn–10Mn	/	∼70	/	/	/	∼160	/	/	[143]
Zn–15Mn	/	90	/	/	/	178	/	/	[143]
Zn–20Mn	/	81	/	/	/	130	/	/	[143]
Zn–2Fe	/	76.27	151.2	181.9	8.5	106.34	/	/	[87]
Zn–2Fe–2Mn	/	87.74	186.2	226.8	15.9	135.98	/	/	[87]
Zn-0.8Li-0.1 Mg	/	148.10	358.50	460.78	1.39	/	/	76.55	[88]
Zn-0.6Li-0.5 Mg	99.45	163.49	199	345	0.8	/	/	75.6	[145]
**Zn-based composites**									
Zn-0.5SiC	/	∼46	/	/	/	∼45	/	/	[17]
Zn–1SiC	/	∼52	/	/	/	∼75	/	/	[17]
Zn–2SiC	/	74.1	/	/	/	121.8	/	/	[17]
Zn–3SiC	/	∼80	/	/	/	∼45	/	/	[17]
Zn-0.1RGO	/	/	111.3	148.5	11.7	/	/	/	[18]
Zn-0.2RGO	/	/	142.9	182.1	14.1	/	/	/	[18]
Zn-0.3RGO	/	/	115.7	155.2	12.9	/	/	/	[18]
Zn–TiC@0.2RGO	97.1	/	153.6	227.2	14.9	/	/	/	[90]
Zn-CNTs	/	73	132	175	13.8	171	/	7.2	[19]
Zn-CNTs@Ag	/	87	178	221	16.1	208	/	10.1	[19]
Zn–CNF	/	69.4	122.4	180.2	8.1	/	/	/	[91]
Zn–La@CNF	/	80.3	158.3	243.4	9.3	/	/	/	[91]

a Abbreviation: Elongation (E, %), Elastic modulus (E, GPa).

Additionally, as shown in Table 2, the mechanical behaviors of scaffolds are not solely dependent on material composition but can also be effectively controlled through structural design [132,133]. The strength and elastic modulus of scaffolds can be predicted by the Gibson and Ashby equation, as shown in Eqs. (8), (9). As demonstrated in Eq. (10), the mechanical behaviors of scaffolds can be effectively controlled by controlling their porosity [[134], [135], [136]]. Liu et al. found that low porosity of scaffolds meant higher strength and elastic modulus. The CS and E of Zn-0.8Li-0.1Mg scaffold with a porosity of 80 % was 11.89 and 313.58 MPa, lower than 41.04 and 966.64 MPa of the scaffold with a porosity of 60 %. The struct thickness and the area of material at the cross sections decreased with increasing porosities, thus deteriorating the resistance to load and deformation [88]. Besides porosity control, the choice of unit cells significantly influences the mechanical behavior of scaffolds [137,138]. Lietaert et al. observed that porous scaffolds with varying unit cells exhibit distinct mechanical properties. For example, the plateau stress of the scaffold with a dodecahedron unit was 13 MPa, while the plateau stress of the scaffold with a diamond unit with the same porosity was 10 MPa [98].

(8) $$\frac{\sigma_{p}}{\sigma_{s}}=C_{1}{\left(\frac{\rho_{p}}{\rho_{s}}\right)}^{n}$$

(9) $$\frac{E_{p}}{E_{s}}=C_{2}{\left(\frac{\rho_{p}}{\rho_{s}}\right)}^{n}$$

(10) $$Porosity=\left(1‐\frac{\rho_{p}}{\rho_{s}}\right)\times 100$$

where *σ*_*s*_, *E*_*s*_, *ρ*_*s*_ are the yield strength, elastic modulus and density of the bulk materials. *σ*_*p*_, *E*_*p*_, *ρ*_*p*_ are the yield strength, elastic modulus and density of scaffolds. *C*_*1*_, *C*_*2*_ and *n* are constant coefficients and exponents.

**Table 2:** Mechanical properties of Zn-based BM scaffolds fabricated by L-PBF.

Composition (wt.%)	Structure design			Mechanical properties^a^			References
	Type of unit	Porosity (%)	Structure parameters^※^ (mm)	CYS (MPa)	CS (MPa)	E (GPa)	
Zn	Diamond	67	PS 0.6; ST 0.4	10.8	/	0.786	[80]
Zn	Diamond	81.1	PS 0.7; ST 0.3	4.2	/	0.4	[21]
		74.3	PS 0.6–0.8; ST 0.4–0.2	∼6.5	/	∼0.45	[21]
		67.4	PS 0.6; ST 0.4	10.8	/	0.786	[21]
Zn	Diamond	67	PS 0.6; ST 0.4	12.7	22.9	0.95	[116]
Zn	/	75.8	ST 0.5	11.7	/	0.763	[18]
Zn	Gyroid	67	US 2	11.99	18.27	0.494	[23]
Zn	Diamond	/	PS 0.4; ST 0.6	14.9	22.9	0.95	[22]
Zn	IWP	70	US 2	6.01	11.14	0.303	[88]
Zn–1Mg	Diamond	67	PS 0.6; ST 0.4	/	40.9	1.17	[14]
Zn–2Mg				/	35.3	1.34	[14]
Zn–5Mg				/	23.6	1.02	[14]
Zn-0.5 Mg	BCC	66	US 1; ST 0.287	10.8	14.7	0.358	[83]
Zn–3Mg	Gyroid	67	US 2	48.3	50.52	2.37	[104]
Zn-2WE43	Diamond	67	PS 0.6; ST 0.4	50.9	60.5	1.91	[116]
Zn-5WE43				66.2	73.2	2.48	[116]
Zn-8WE43				50.9	50.9	2.54	[116]
Zn-0.7Li	Gyroid	80	US 2	/	18.2	0.298	[15]
Zn–7Al	/	∼78.46	PS 0.8	100	150	/	[146]
Zn–7Al-0.5Sn				∼125	∼190	/	[146]
Zn–7Al–1Sn				∼150	∼210	/	[146]
Zn–7Al–2Sn				180	325	/	[146]
Zn–7Al–3Sn				∼170	∼300	/	[146]
Zn–3Mg-0.5Ag	/	/	PS 0.8	/	375	/	[86]
Zn–3Mg–1Ag				/	∼325	/	[86]
Zn–3Mg–2Ag				/	273	/	[86]
Zn–3Mg	Gyroid	67	US 2	40.75	41.45	2.01	[23]
Zn–3Mg–2Cu				41.15	42.35	2.16	[23]
Zn-0.1RGO	/	75.8	ST 0.5	∼14	/	∼0.90	[18]
Zn-0.2RGO				19.1	/	1.125	[18]
Zn-0.3RGO				∼15	/	∼1	[18]
Zn-0.8Li-0.1 Mg	IWP	60	US 2	39.44	41.04	0.967	[88]
		70		23.29	24.59	0.649	[88]
		80		11.38	11.89	0.314	[88]

a Abbreviation: Unit size (US), Strut thickness (ST), Pore size (PS).

Mechanical behaviors can be evaluated by many indicators, such as tensile strength, compressive strength and elastic modulus as discussed above. However, when considering implants serving in corrosive environments and bearing continuous stress stimulation, the evaluation of corrosion fatigue properties is indispensable. Li et al. reported the corrosion fatigue behavior of porous pure Zn. In the air, Zn scaffold had high relative fatigue strength (0.7 σ_y_). In r-SBF, the fatigue strength was increased to 0.8 σ_y_ due to the accumulation of corrosion products. The topological design could determine the fatigue behavior of scaffolds. Compared with specimens with uniform design, samples with functionally graded structure had higher fatigue strength [139]. Zhao et al. had the investigation on the corrosion fatigue behavior of Zn–Mg alloy. Zn–3Mg scaffolds had a 227 % higher fatigue strength, but a 17 % lower strain accumulation at 10^6^ cycles than that of pure Zn counterparts, explained by the grain refinement, dislocation pile-up and the uniformly distributed stress of the gyroid structure [104].

The mechanical strength of bulk materials fabricated through conventional manufacturing methods can be effectively enhanced by alloying and doping reinforcement particles, as discussed in Section 2.3. The UTS of the hot-extruded Zn-0.8Li-0.4 Mg alloy reached 646.69 MPa, far higher than 166.79 MPa of hot-extruded Zn [31]. However, the high elastic modulus of these materials cannot be significantly reduced through compositional optimization, resulting in stress shielding effect. For Zn-based BMs fabricated by L-PBF, alloying also significantly enhanced the strength. Zn-0.8Li-0.1Mg alloy had a UTS of 460.78 MPa, higher than 134.03 MPa of Zn by L-PBF [88]. Notably, the strength of pure Zn by L-PBF was lower than that by hot-extrusion mainly due to the existence of unavoidable micro pores, as discussed in Section 4.3 and the inhomogeneous microstructure resulted from layer-upon-layer deposition. In addition to composition optimization, structure design of scaffolds fabricated by L-PBF can efficiently modulate the mechanical properties of the porous implants. The incorporation of pores notably reduces the modulus of the scaffold. The elastic modulus of the porous Zn-0.7Li scaffolds with a gyroid unit and a porosity of 80 % was 298.0 MPa, comparable to those of cancellous bone [15]. Furthermore, by adjusting parameters such as pore shape and porosity, precise modulation over the strength and modulus of the scaffolds can be achieved. This allows for the design and manufacture of implants with individualized mechanical properties for specific patient [133].

### 4.8 Degradation behavior

**Fig. 9:**
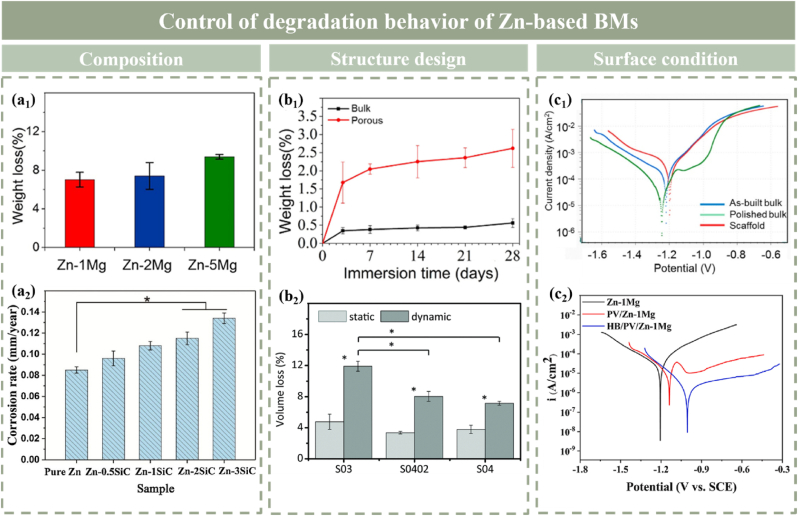
Control of degradation behavior of Zn-based BMs: Composition: (a_1_) weight loss of Zn–Mg alloy after immersion tests [14], (a_2_) corrosion rate of Zn–SiC composites [17]; Structure design: (b_1_) weight loss of bulk and porous Zn-0.7Li after immersion tests [15], (b_2_) volume loss of pure Zn scaffolds with different structure designs [21]; Surface condition: (c_1_) PDP curve of bulk and porous Zn-0.7Li [15], (c_2_) PDP curve of Zn–1Mg scaffolds with coating [84].

The degradation behavior of Zn-based BMs fabricated by L-PBF is primarily determined by composition, structural design, and surface condition. Regarding composition, the microstructure characteristics, such as grain size, phase composition, and the composition of degradation products vary among metals with different composites, consequently resulting in distinct degradation behaviors. For instance, the degradation rate of Zn–Ag alloy increased with the increase of Ag content. The degradation rate of Zn–8Ag was 0.133 mm/year, much higher than 0.081 mm/year of pure Zn [127]. The corrosion resistance of Zn–Mg alloy decreased with the increase of alloying element content, as shown in Fig. 9a_1_. The *in vivo* tests show that the structural integrity of pure Zn and Zn–1Mg scaffolds was kept after 12 weeks of implantation [14]. The precipitated secondary phase and Zn matrix formed micro-galvanic cells, greatly reducing the corrosion resistance of Zn alloys [147]. Shuai et al. found that the degradation rate of Zn–3Ce decreased from 0.0336 mm/year of pure Zn to 0.0242 mm/year, and the same trend was also obtained by adding Nd. During the corrosion process of Zn, dense Zn chloride formed on the surface, affected by carbonate ions to form Zn carbonate with amorphous state. The addition of rare earth elements can effectively restrict ion transfer, improving the stability of Zn chlorides, thus improving the corrosion resistance [16,142]. As for Zn-based composites, the degradation rate of Zn–3SiC was 0.134 mm/year, 57.6 % higher than that of pure Zn, as shown in Fig. 9a_2_ [17]. The corrosion rate of Zn-0.2RGO was 0.27 mm/year, 145.5 % higher than that of pure Zn [18]. The formation of micro-galvanic cells between nanoparticles and the Zn matrix in local areas contributes to accelerated corrosion [17,18].

Structural design markedly influences the degradation behavior of Zn-based BMs. Through topological design, it is possible to regulate the specific surface area and permeability of scaffolds, which in turn significantly impacts their degradation rates. Qin et al. reported the degradation behavior of Zn-0.7Li bulk material and scaffolds. After 28 days of immersion test, the weight loss of the scaffold was 2.6 %, while the weight loss of the bulk was only 0.56 %, as shown in Fig. 9b_1_. The porous scaffolds had a larger specific surface area, indicating a larger surface area per volume exposed to the immersion medium, thus resulting in an accelerated corrosion compared with bulk materials [15]. Li et al. designed porous scaffolds with different porosity distributions, and the degradation rate increased with the increase of porosity, as shown in Fig. 9b_2_. When the porosity was 81.1 %, the volume loss of the scaffold was 11.39 %. While, at a porosity of 67.4 %, the volume loss was reduced to 7.1 %. Structure design could change the permeability of scaffolds, and higher porosity meant higher permeability, thus accelerating the degradation [21].

Ultimately, surface condition plays a pivotal role in influencing degradation behaviors. It is generally observed that an increase in surface roughness often corresponds to an elevated corrosion rate. Compared with smooth surface, high surface roughness increases the surface area exposed to the corrosion medium, thus resulting in an increasing corrosion rate. Moreover, the aggressive ions infiltrate into grooves of the surface with high surface roughness, contributing to the initiation of pitting corrosion, accelerating the corrosion [148,149]. Qin et al. reported the effect of surface roughness on the corrosion rate of Zn-0.7Li by L-PBF (Fig. 9c_1_). The attached powders on the surface of as-built Zn-0.7Li alloys increased the surface roughness. After polishing, the corrosion current density of the bulk material decreased to 28.5 μA/cm^2^ from 101.0 μA/cm^2^ of as-built samples [15]. The coating on the surface can also effectively control the degradation rate of Zn-based BMs. As reported by Zhang et al., HB/PV/Zn–1Mg scaffolds exhibited a significantly decreased corrosion current density (1.873 μA/cm^2^) compared with Zn–1Mg scaffold (19.561 μA/cm^2^), as shown in Fig. 9c_2_ which was resulted from the effect of physical barrier [84].

As discussed in Section 2.2, the degradation behavior of bulk Zn-based BMs produced through conventional manufacturing methods is typically modulated using composition optimization. For Zn-based BMs fabricated by L-PBF, both compositional and structural design enable modulation of degradation behavior. For example, the corrosion rate of Zn (0.18 mm/year) decreased to 0.14 mm/year after alloying with 1 wt% Mg [82]. However, the ability to modulate degradation behavior through composition adjustments is quite limited. Structural design offers a more effective method for controlling degradation behavior. Porous scaffolds exhibit a higher degradation rate due to their increased specific surface area compared to bulk materials. After 28 days of *in vitro* immersion, the weight loss of a Zn-0.7Li scaffold was 2.6 %, while the weight loss of the bulk material was only 0.56 % [15]. By adjusting structural design parameters, such as pore size and cell type, the degradation behavior of porous scaffolds can be precisely regulated. Hence, L-PBF provides the opportunity to manufacture orthopedic implants with customized degradation behaviors.

### 4.9 Biocompatibility

**Fig. 10:**
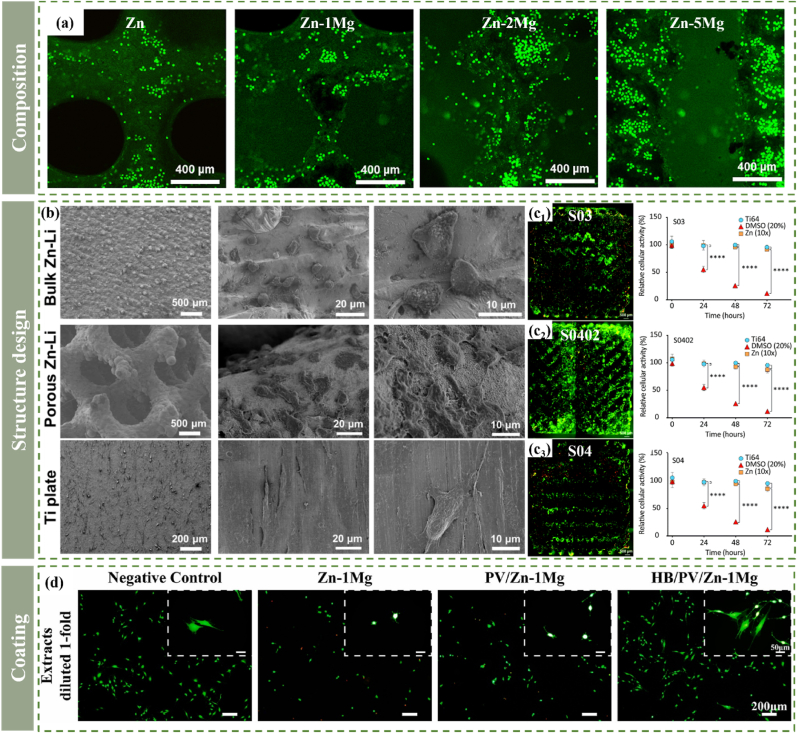
Biocompatibility of Zn-based BMs by L-PBF: (a) live/dead fluorescent imaging of MC3T3-E1 cells cultured on pure Zn and Zn–Mg alloy scaffolds for 1 day [14]; (b) MC3T3-E1 cell morphology on the surface of Ti, as-built bulk, and porous Zn-0.7Li alloy [15]; (c) images of fluorescent MG-63 cells cultured with samples for 1 day in cross-sections of scaffolds and relative cellular activities of cells upon exposure to Zn scaffolds with different structures [21]; (d) live/dead staining of MC3T3-E1 cells co-cultured with extracts (diluted 1-fold) from scaffolds with different coatings for 1 day [84].

Li et al. reported the cytocompatibility of L-PBF pure Zn scaffolds. MG-63 cells were evenly distributed around and within Zn scaffolds. After 24 h of seeding, most of the cells were viable and attached to the surface of the scaffold. The cell morphology on the surface of Zn scaffolds was comparable to that of TC4 alloy [80]. Lietaert et al. investigated the cytocompatibility of pure Zn scaffolds, while the result was completely different from that reported by Li et al. After the perfusion bioreactor culture for 13 days, neo-tissue was found to form on the surface of TC4 alloy, while there was no cell attachment and growth on Zn scaffolds. The low lactate production rate and DNA measurements all proved that the cell viability on the Zn scaffolds was low. The considerable variation in outcomes could be attributed to the distinct cell types, surface treatments, and immersion media employed [98]. To improve biocompatibility of L-PBF Zn-based BMs by L-PBF, composition optimization, structure design and surface modification have been successfully utilized. The composition notably influences biocompatibility. The cell viability of Zn–Mg scaffolds was shown in Fig. 10a. Compared with pure Zn, MC3T3-E1 cells on Zn–Mg scaffolds were significantly visibly more and larger, implying better cell viability. It was reported that a high concentration of Zn^2+^ (23.9 mg/L) led to the bad cell viability of MC3T3-E1. While, when the concentration of Zn^2+^ was 12.1 mg/L, the cells showed a healthy shape, and were well spread and proliferated. For pure Zn, the concentration of Zn^2+^ in the extract reached 23.2 mg/L. The relatively high concentration explained the low biocompatibility. For Zn–Mg alloy, the release of Mg^2+^ significantly improved the cytocompatibility. The concentration of Mg^2+^ in extracts increased with the increasing Mg content, indicating the increased biocompatibility [14,150]. Shuai et al. studied the biocompatibility of Zn–Cu alloys. The cell viability of Zn–3Cu alloy was higher than that of pure Zn, indicating that Cu^2+^ promoted cell proliferation [85]. As for doping reinforcements, it was found that the addition of RGO could improve the biocompatibility. On one hand, RGO contained many oxygen-containing functional groups, which could interact with cell membrane phospholipids and proteins, resulting in better cell adhesion and growth compared with pure Zn. On the other hand, RGO could lead to changes in gene expression regulated by non-coding RNA in the cytoplasm, which promoted the differentiation of stem cells [18]. Gao et al. added nano-SiC particles to pure Zn, and the nanocomposites showed good biocompatibility. While, the biocompatibility of the composites with different amounts of nano-SiC particles showed no significant difference. SiC had good biocompatibility and could promote cell proliferation and osteogenic differentiation. However, the addition of nano-SiC accelerated the degradation rate, and the excessive release of Zn^2+^ was harmful to cell proliferation. Consequently, taking into account these two factors, the addition of nano-SiC exhibited a negligible effect on the viability of MG-63 cells [17].

Besides composition, structural design significantly influences the biocompatibility of materials [151,152]. Qin et al. reported the cytocompatibility of Zn-0.7Li bulk and scaffolds. As shown in Fig. 10b, the cells on the surface of Zn-0.7Li bulk were shrunk in a spherical shape, indicating a relatively poor cytocompatibility. While, the cells had a healthier morphology on the surface of porous Zn-0.7Li scaffold. The better cytocompatibility was related to the curvature of the surface and the larger specific surface area of designed scaffolds [15]. Li et al. designed and manufactured porous Zn scaffolds with porosity of 81.1 % (S03), 67.4 % (S04) and 67.4–81.1 % (S0402) respectively, and studied their biocompatibility. The results of the direct contact cytocompatibility test showed that, after 24 h of contacting with porous scaffold, most MG-63 cells were viable, and only a small number of cells showed compromised membrane integrity. As shown in Fig. 10c, compared with S03, S04 and S0402 samples contained more adherent cells, explained by the large strut surface area for cell-surface interactions provided by small pores and cell accumulation from the pore-clogging [21]. Ultimately, surface modification markedly improves the biocompatibility of Zn-based BMs. It was reported that HB/PV/Zn–1Mg group exhibited higher cell viability and better cell morphology compared with Zn–1Mg group, as shown in Fig. 10d, due to the lower Zn^2+^ concentration and addition of BMP2, which was effective to promote biocompatibility [84].

As discussed in Section 2.1, the biocompatibility of bulk Zn-based BMs fabricated by conventional manufacturing methods is majorly optimized by composition design. For instance, adding Li can inhibit the release of high concentrations of Zn^2+^ and promote the release of Li^+^, which is beneficial for improving biocompatibility [52]. Compared to bulk materials, porous scaffolds fabricated by L-PBF have a larger specific surface area and release more Zn^2+^, potentially reducing biocompatibility [152]. However, by designing the structure to regulate specific surface area and permeability, the amount of ion release can be optimized to enhance biocompatibility. Additionally, optimizing the pore size and surface curvature of these porous scaffolds can create favorable conditions for cell adhesion, proliferation, and other behaviors, further enhancing biocompatibility [151].

### 4.10 Bioactive functions

**Fig. 11:**
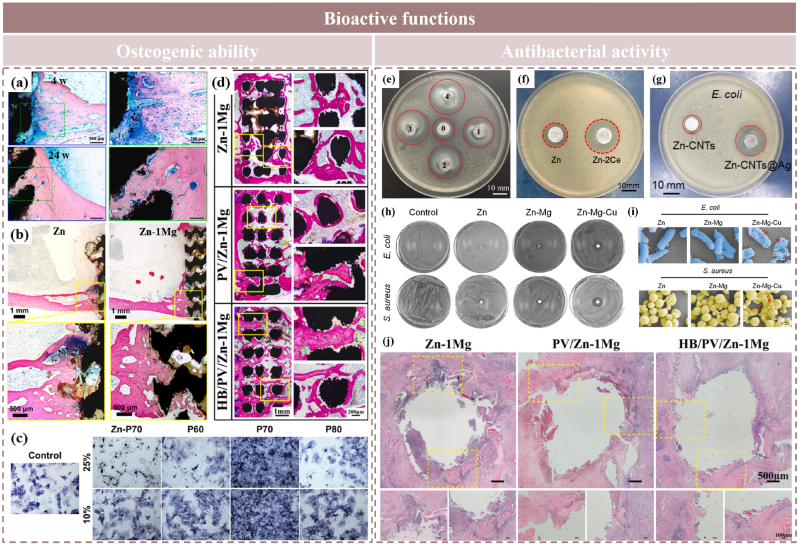
Bioactive functions of L-PBF Zn-based BMs: Osteogenic ability: Hard tissue sections of methylene staining: (a) pure Zn scaffold (implanted in New Zealand rabbits for 4 and 24 weeks) [22]; (b) Zn and Zn–1Mg alloy scaffolds (implanted in New Zealand rabbits for 12 weeks) [14]; (c) ALP results of Zn-0.8Li-0.1 Mg scaffolds with different porosities (60 %, 70 %, and 80 %) [88]; (d) hard tissue slices of coated Zn–1Mg scaffolds (implanted in rats for 8 weeks) [84]; Antibacterial activity: Inhibition zones formed around (e) Zn-xCu (x = 0, 1, 2, 3 and 4) (*E. coli*) [85], (f) Zn–2Ce alloy (*E. coli*) [16], (g) Zn-CNTs@Ag composites (*E. coli*) [127], (h) Zn–Mg and Zn–Mg–Cu alloys (*E. coli* and *S. aureus*) [23]; (i) the morphology of bacteria (*E. coli* and *S. aureus*) incubated with 25 % alloy extracts [23]; (j) H&E staining slices of the infected (*S. aureus*) SD rat femoral condyle after the implantation of coated Zn–1Mg scaffolds for 7 days [84].

Research on L-PBF Zn-based BMs, particularly for orthopedic implants, primarily focuses on osteogenic activity and antibacterial properties as key bioactive functions. Xia et al. conducted a study on the ALP activity of pure Zn scaffolds. The osteogenic differentiation was promoted in the 10 % pure Zn scaffold extract. The *in vivo* osteogenesis was also evaluated as shown in Fig. 11a. With the time passing by, the bone density around the scaffold increased, indicating that the bone tissue gradually matured. At week 4, a fibrous tissue layer was observed between the bone tissue and scaffold. After 12 weeks of implantation, the new bone was in close contact with the implant, and the new bone grew into the scaffold. At week 24, more volume of new bone grew into the pores of scaffold and closely connected to the scaffold. Studies showed that porous pure Zn scaffolds had favorable osteogenic ability [22]. The osteogenic activity of Zn-based BM scaffolds can be enhanced through the optimization of composition, structure design, and surface modification. Regarding composition, Qin et al. investigated the osteogenic ability of porous Zn and Zn–1Mg scaffolds by implanting them into the femur of rabbits, as illustrated in Fig. 11b. At week 6 after implantation, fibrous tissue layers between scaffolds and bone tissue were observed in both pure Zn and Zn–1Mg. At week 12, the fibrous tissue layer still existed in pure Zn group. While, Zn–1Mg scaffold was in tight contact with the surrounding bone. Moreover, more new bone formed in the Zn–1Mg scaffold, indicating better osseointegration. The excessive release of Zn^2+^ could lead to the lack of direct contact between the new bone and the pure Zn implant, resulting in poor osseointegration. For Zn–1Mg alloy, the Mg rich phase was preferentially corroded after implantation to inhibit the release of Zn^2+^, thus improving the osseointegration at the early stage of implantation [14]. Zhao et al. fabricated pure Zn, Zn–Mg and Zn–Mg–Cu scaffolds and investigated their osteogenic abilities *in vitro* and *in vivo*. ALP activity of Zn–Mg and Zn–Mg–Cu alloy was higher than that of pure Zn. The higher production levels in terms of proteins related to osteogenesis were observed in Zn–Mg and Zn–Mg–Cu groups. The *in vivo* tests showed there was more amount of new bone in Zn–Mg and Zn–Mg–Cu groups, indicating better osteogenic capacity compared with pure Zn [23]. In terms of structural design, Liu et al. assessed the *in vitro* osteogenic ability of Zn-0.8Li-0.1Mg scaffolds with varying porosities, as shown in Fig. 11c. These scaffolds, differing in porosity, exhibited distinct structural characteristics such as surface area and permeability. These characteristics significantly affected ion release during degradation, thus directly influencing the *in vitro* osteogenic activity of implants. The scaffolds with a porosity of 70 % exhibited the best osteogenic ability, attributed to the lowest content of released Zn^2+^ and a moderate content of released Li^+^ [88]. As for surface modification, Zhang et al. realized the improvement of osteogenic ability of Zn alloy scaffolds by surface modification. As shown in Fig. 11d, the *in vivo* osteogenic activity of coated scaffolds was greatly enhanced, explained by the loading of BMP2 and low concentration of Zn^2+^. Moreover, after surface modification, the wetting angle of scaffolds decreased a lot, helpful for osteoblast adhesion, migration and functionalization, thus further promoting osseointegration [84].

Regarding the antibacterial ability, Shuai et al. reported the antibacterial activity of Zn–Cu alloys, as shown in Fig. 11e. Inhibition zones were observed in all samples, indicating a certain antibacterial ability of pure Zn and Zn–Cu alloys. Compared with pure Zn, Zn–Cu groups exhibited a larger inhibition zone, implying the stronger antibacterial activity, resulted from the release of Cu ions [85]. Yang et al. found that Zn–2Ce alloy showed enhanced antibacterial effect, reflected by the larger inhibition zone diameter (8.12 mm) compared with 4.37 mm of pure Zn (Fig. 11f) [16]. The addition of Ag, which showed a broad-spectrum antibacterial function, also significantly increased the antibacterial ability of Zn-based composites (Fig. 11g) [127]. Zhao et al. investigated the antibacterial activity of Zn–Mg–Cu alloy. As shown in Fig. 11h–i, Zn–Mg–Cu showed the largest inhibition zone compared with pure Zn and Zn–Mg alloys. *E. coli* and *S. aureus* in irregular shapes with rough and shriveled surfaces were observed in Zn–Mg–Cu group. The results showed that Zn–Mg–Cu alloy had the strongest antibacterial activity [23]. Surface modification is also effective to enhance antibacterial ability. As shown in Fig. 11j, only a small amount of inflammatory cell infiltration and no obvious sequestrum were found in PV/Zn–1Mg and HB/PV/Zn–1Mg groups, indicating the controlled *S. aureus*-induced infection, due to the load of Van, which had strong antibacterial activity [84].

The bioactive functions of BM implants are primarily determined by the type and amount of ions released. As discussed in Section 2.4, in bulk materials fabricated by conventional manufacturing methods, types and proportions of alloying elements or doped materials have been changed to modulate the bioactive function of the implant. Composition optimization can also endow materials fabricated by L-PBF with designed bioactive functions. Moreover, the structural design freedom offered by L-PBF allows for the fabrication of scaffolds with varying porous structures, resulting in different degradation behaviors, distinct metal ion releases, and consequently diverse osteogenic and antibacterial activities. Therefore, L-PBF is anticipated to enable the fabrication of scaffolds with customized bioactive functions tailored to specific clinical needs, thereby advancing precision medicine.

## 5 Conclusions and outlook

In this paper, the research status of L-PBF Zn-based BMs, including pure Zn, Zn alloys and Zn-based composites, is reviewed. Powder particles, structure design, processing optimization, chemical compositions, surface modification, microstructure, mechanical properties, degradation behavior, biocompatibility and bioactive functions are discussed and analyzed. By optimization of powder particle composition and structure design, along with surface modification, Zn-based BM scaffolds with sufficient mechanical strength, proper degradation behaviors, satisfied biocompatibility and bioactive functions can be fabricated successfully by L-PBF. The detailed conclusions are as follows.

(1) Zn-based BM bulk and scaffolds have been successfully fabricated by L-PBF. Extremely high fusion quality (RD >99.5 %) of Zn-based BMs can be achieved by utilizing high-quality powders, controlling of fume and spatter, and optimizing the processing parameters. The dimensional accuracy of scaffolds is influenced by the heat input and can be further improved through surface post-treatment. Notably, the chemical composition of L-PBF Zn alloys tends to deviate from the original powder composition, owing to varying evaporation tendencies of different elements during the L-PBF process.
(2) The comprehensive performance of Zn-based BMs can be enhanced through composition optimization of powder particles. Alloying and doping significantly improve the mechanical properties of Zn-based BMs, with Mg and Li exhibiting the best strengthening effects. The Zn-0.8Li-0.1Mg alloy demonstrates a remarkable tensile strength of 460.78 MPa, the highest among all reported L-PBF Zn alloys. The degradation behavior of Zn-based BMs is modifiable by incorporating varying types and amounts of alloying elements or nanoparticles. In terms of biocompatibility, better biocompatibility can be achieved through the addition of Mg and Cu elements by controlling the release of metal ions. The addition of nanoparticles, such as RGO, also helps to enhance biocompatibility. Regarding bioactive functions, the addition of Mg elements can significantly enhance the osteogenic ability of Zn-based BMs. The addition of Cu, Ag greatly improves the antibacterial activity.
(3) Structure design plays a pivotal role in determining scaffold's performance. Zn-based BM Scaffolds, varying in unit types and porosities, demonstrate distinct mechanical behaviors. Notably, an increase in porosity leads to a reduction in both strength and elastic modulus of the scaffolds. Compared to bulk materials, porous scaffolds possess a higher specific surface area and permeability, which results in accelerated degradation rates. Furthermore, distinct structure designs have different structural characteristics, such as pore size and surface curvature, thus exhibiting different cytocompatibility.
(4) A drug-loaded composite coating was successfully prepared on the surface of L-PBF Zn–1Mg scaffolds. The coating mitigated the excessive release of Zn^2+^ and offered a surface more favorable for osseointegration. The reduced Zn^2+^ release, coupled with the existence of BMP2 in the coating, enhanced both the biocompatibility and osteogenic activity of the scaffolds. Additionally, the inclusion of vancomycin in the drug-loaded coating substantially improved the antibacterial activity.
(5) L-PBF offers significantly more structural design freedom compared to conventional manufacturing methods, enabling the reliable fabrication of porous scaffolds with individualized structures. These porous scaffolds exhibited a lower elastic modulus and a higher degradation rate compared to bulk materials. Effective regulation of mechanical properties, degradation behavior, biocompatibility, and bioactive functions of scaffolds can be achieved through material–structure–function integrated design. This capability allows for the fabrication of customized implants with target performance for patient-specific needs.

Considering the current state of research, future developments in L-PBF of Zn-based BMs should focus on the following key aspects.

(1) Powder particle composition optimization. Given the specific requirements for medical applications, the development of Zn-based BMs with further enhanced mechanical strength, appropriate degradation behavior, improved biocompatibility, and multifunctional bioactive functions, such as high angiogenesis and anti-cancer activities, is imperative. Machine learning offers a promising avenue for rapidly predicting material properties and identifying material compositions that align with optimal characteristics. Concurrently, the design of powder compositions for L-PBF needs significant consideration. In the L-PBF process, the evaporation and burning of elements lead to changes in the chemical composition of the fabricated samples, thereby directly influencing their properties. Therefore, the loss of elements should also be considered in the alloying design, ensuring the desired chemical composition of the fabricated components.
(2) Structure design optimization. The structure design of Zn-based BM scaffolds critically influences their overall performance. Yet, the impact and fundamental mechanisms through which structural design affects the degradation behavior and biocompatibility of Zn-based BMs *in vivo* remain poorly understood. These insights, crucial for the design of scaffolds with customized performance, necessitate thorough investigation. Furthermore, to precisely control the mechanical and biodegradable properties of implants, machine learning and topology optimization can be employed to optimize the structural design of scaffolds.
(3) Surface modification. The development of surface modifications for L-PBF Zn-based BM scaffolds is still at an early stage. The preparation of coatings, such as hydrogels, on scaffold's surfaces shows promise. These coatings can significantly mitigate the release of excess Zn^2+^, thereby substantially improving biocompatibility. Moreover, these coatings can serve as drug carriers, endowing the scaffolds with more bioactive functions.
(4) Interaction mechanism of L-PBF Zn-based BM implants and the body. The early healing phase is decisive to the speedy and successful recovery of the damaged tissue or organs. The change of biological and mechanical response resulting from the degradation of implants significantly influences the early healing phase. For instance, it is found that the concentration of dissolved Zn^2+^ and some other alloying element ions are directly related to bone regeneration and antibacterial ability. However, the underlying biological regulatory mechanisms, particularly immunological aspects, remain largely unexplored. Established biomechanical principles, drawn from bioinert porous scaffolds like titanium alloys, inform the reasonable structure design of medical implants. While, for biodegradable Zn-based BM implants, the combination of biological factors regulated by the dissolved ions and mechanical factors contributed by the geometrical structure have to be considered simultaneously. L-PBF provides the flexibility to the fabrication of customized structures. Unraveling the regulation mechanism of how structure design influences tissue healing is the key to take advantage of such a flexibility and transform it to precise therapies eventually.
(5) Exploration of more medical applications. Presently, the research on L-PBF Zn-based BMs primarily targets orthopedic implants. However, there is a growing need for L-PBF in the fabrication of clinical devices that demand complex structural designs, such as cardiovascular stents, GBR membranes, interbody fusion devices, and biodegradable energy devices. In future developments, Zn-based BMs can be designed and fabricated using L-PBF for a wider range of medical applications, taking into account their specific functional requirements. It is imperative to conduct thorough investigations of their comprehensive properties both *in vitro* and *in vivo* to ensure their effectiveness and safety in medical use.

## Ethics approval and consent to participate

This review article does not require any ethical approval or allied consents for publication.

## CRediT authorship contribution statement

**Aobo Liu:** Writing – original draft, Conceptualization. **Yu Qin:** Writing – original draft. **Jiabao Dai:** Writing – original draft. **Fei Song:** Writing – original draft. **Yun Tian:** Writing – review & editing, Supervision. **Yufeng Zheng:** Writing – review & editing, Supervision, Funding acquisition. **Peng Wen:** Writing – review & editing, Supervision, Funding acquisition.

## Declaration of competing interest

Yufeng Zheng is an editor-in-chief for *Bioactive Materials* and was not involved in the editorial review or the decision to publish this article. Peng Wen is an editorial board member for *Bioactive Materials* and was not involved in the editorial review or the decision to publish this article. All authors declare that there are no competing interests.
